# TDP-43 Coincides with Noradrenergic Neurons in Alzheimer’s Disease (AD) and Primary Age-Related Tauopathy (PART)

**DOI:** 10.3390/cells15201850

**Published:** 2026-10-12

**Authors:** Gokhan Uruk, Hossam Youssef, Rodolfo G. Gatto, Nadia Hossain, Jerusha Bhaskaran, Yuqi Tian, Mary M. Machulda, Ronald C. Petersen, Jennifer L. Whitwell, Robert Ross Reichard, Keith A. Josephs

**Affiliations:** 1 Department of Neurology, Mayo Clinic, Rochester, MN 55905, USA; uruk-contreras.gokhan@mayo.edu (G.U.); youssef.hossam@mayo.edu (H.Y.); gatto.rodolfo@mayo.edu (R.G.G.); hossain.nadia@mayo.edu (N.H.); bhaskaran.jerusha@mayo.edu (J.B.); peter8@mayo.edu (R.C.P.); 2 Department of Radiology, Mayo Clinic, Rochester, MN 55905, USA; tian.yuqi@mayo.edu (Y.T.); whitwell.jennifer@mayo.edu (J.L.W.); 3 Department of Neuropsychiatry (Neuropsychology), Mayo Clinic, Rochester, MN 55905, USA; machulda.mary@mayo.edu; 4 Department of Laboratory Medicine and Pathology (Neuropathology), Mayo Clinic, Rochester, MN 55905, USA; reichard.robert@mayo.edu; 5 Department of Neurology, Behavioral Neurology, and Movement Disorders, Mayo Clinic, College of Medicine, and Science, 200 1st Street Southwest, Rochester, MN 55905, USA

**Keywords:** Alzheimer’s disease (AD), primary age-related tauopathy (PART), Lewy body disease (LBD), Artificial Intelligence (AI), TAR DNA-binding protein 43 (TDP-43), noradrenergic, glutamatergic, cholinergic

## Abstract

**Highlights:**

**What are the main findings?**

- TDP-43 mapping across the amygdala, hippocampal subfields, and entorhinal cortex (ERC) reveals preferential accumulation in neuromodulatory noradrenergic neurons.
- Cell-type colocalization analysis shows limited involvement of glutamatergic terminals and cholinergic neurons, with LBD consistently reducing pTDP-43 burden across transmitter systems.

**What is the implication of the main finding?**

- Neuromodulatory circuit vulnerability suggests that noradrenergic pathways represent a major convergence point for AD-related molecular stressors.
- Disease-specific cellular signatures indicate that coexisting synucleinopathy shapes TDP-43 cellular targeting and may contribute to limbic network dysfunction.

**Abstract:**

TAR DNA-binding protein 43 (TDP-43) pathology is a major contributor to cognitive decline in Alzheimer’s disease (AD), yet the cellular substrates and circuit-level consequences of its limbic accumulation remain poorly defined. Here, we combined multiplex immunofluorescence with 3D Artificial Intelligence (AI)-based object detection and quantitative colocalization to map phosphorylated TDP-43 (pTDP-43) across neuronal subtypes in the amygdala, hippocampal subfields, and entorhinal cortex (ERC) in human AD, AD with Lewy body disease (AD-LBD), and primary age-related tauopathy (PART). Across 15 deeply phenotyped cases, pTDP-43 showed preferential accumulation in neuromodulatory noradrenergic neurons, with more limited involvement of glutamatergic terminals and cholinergic populations. Noradrenergic neurons marked by dopamine β-hydroxylase (DβH) demonstrated extensive colocalization with pTDP-43 across regions, correlating with APOE genotype, amyloid burden, and tau staging in this cross-sectional cohort, suggesting that neuromodulatory circuits might represent a major convergence point for AD-related molecular stressors. Dopaminergic (tyrosine hydroxylase (TH)) and cholinergic (choline acetyltransferase (ChAT)) neurons showed more restricted but pathology-dependent involvement, with LBD consistently showing lower pTDP-43 burden across multiple transmitter systems. Glutamatergic vesicular glutamate transporter-1 (VGLUT1)-positive neurons and terminals exhibited minimal colocalization except in PART. Principal component (PCA) and hierarchical clustering analyses (HCA) integrating pTDP-43 colocalization with neuropathologic staging metrics (A, B, C, Thal, Braak, LBD) reliably separated PART, AD-TDP, and AD-TDP-LBD groups, with LBD emerging as a dominant modifier of cellular vulnerability. Together, these findings point out that TDP-43 pathology in AD preferentially targets neuromodulatory circuits, is strongly shaped by coexisting synucleinopathy, and provides a cell-type-resolved framework for understanding how TDP-43 contributes to limbic network dysfunction in neurodegeneration.

## 1. Introduction

Alzheimer’s disease (AD) is pathologically characterized by beta-amyloid (Aβ) and hyperphosphorylated tau inclusions often associated with TDP-43 accumulation orchestrating targeted neurodegeneration. TDP-43 proteinopathy defines nearly all cases of amyotrophic lateral sclerosis (ALS) and approximately half of frontotemporal lobar degeneration (FTLD) cases [1]. TDP-43 is also highly prevalent in AD and primary age-related tauopathies (PART), occurring in up to 70% of cases, which some researchers label as limbic-predominant TDP-43 encephalopathy (LATE) [2,3,4,5]. TDP-43 protein is composed of four domains: an N-terminal domain, two RNA recognition motifs (RRM1 and RRM2), and a C-terminal prion-like domain [6,7]. One of the most common post-translational modifications associated with TDP-43 pathological inclusions across the spectrum of sporadic and familial forms of TDP-43 proteinopathies is the phosphorylation of serine residues 409 and 410 (S409/410) of TDP-43 [8]. Clinically, the presence of TDP-43 co-pathology in AD and other diseases is associated with significantly worse outcomes, including accelerated hippocampal atrophy, a lower threshold for dementia, and a more rapid trajectory of cognitive decline compared to patients with AD without TDP-43 co-pathology [2,3,4].

Lewy body disease (LBD) is a frequent neurodegenerative cause of cognitive and neuropsychiatric decline which is defined by the cortical and subcortical accumulation of α-synuclein-rich intraneuronal inclusions, known as Lewy bodies [9]. Individuals with LBD commonly exhibit additional age-related neuropathologies, including extracellular amyloid-β (Aβ) plaques and, in some cases, tau-positive neurofibrillary tangles; thus, it can present itself as a co-pathology to AD [10]. Moreover, TDP-43 exists in 63% of LBD cases [11], and several cohorts show that LBD is found in TDP-43-positive AD cases at rates ranging between 25 and 50% [12].

TDP-43 pathology in AD demonstrates preferential involvement of limbic structures, particularly the amygdala and hippocampus, and follows predictable anatomical progression [2,13]. The sequence of regional TDP-43 screening in AD begins with the amygdala, as this is the earliest site of involvement [13,14,15]. Anatomically, the amygdala is mainly divided into the cortical-like basolateral complex (amygdala-BL), populated by glutamatergic pyramidal projection neurons and diverse GABAergic interneurons, and the striatal-like centromedial complex (amygdala-CM), which is composed almost exclusively of inhibitory GABAergic neurons [16,17,18]. The hippocampus is organized into the dentate gyrus (DG), cornu Ammonis (CA1–CA4) fields, and the subiculum (SUB), comprising distinct circuits of granule cells and pyramidal neurons [19,20,21]. In AD, these structures exhibit early, profound atrophy of comparable magnitude, serving as repositories for misfolded proteins such as Aβ and tau [21,22,23]. This pathology manifests as selective neurodegeneration in hippocampal CA1 and SUB neurons [24,25], while amygdala-BL neurons display aberrant hyperactivity driven by excitation–inhibition imbalances and significant dendritic spine loss [26,27].

Noradrenergic and dopaminergic neurons play central roles in regulating arousal, motivation, and cognitive flexibility. Their selective vulnerability is increasingly implicated in the earliest stages of neurodegeneration [28,29]. Noradrenergic neurons of the locus coeruleus, marked by dopamine β-hydroxylase (DβH) and tyrosine hydroxylase (TH), undergo very early and profound degeneration in AD [30], leading to impaired neuromodulatory tone [31], heightened neuroinflammation [32], and reduced resilience to amyloid-β and tau pathology [33]. Converging evidence suggests that early degeneration of these neuromodulatory systems destabilizes large-scale brain networks, amplifies synaptic vulnerability, and accelerates pathological spread [29,30,31].

Glutamatergic and cholinergic neurons form the core excitatory and modulatory architecture of the mammalian forebrain. Glutamatergic neurons and terminals, identified by markers such as vesicular glutamate transporters (VGLUT1 and 2), sustain long-range communication and synaptic plasticity, though they are among the earliest populations to exhibit excitotoxic stress, dendritic retraction, and thus synaptic loss in the presence of amyloid-β and tau pathology [34,35,36]. In parallel, basal forebrain cholinergic neurons—marked by choline acetyltransferase (ChAT) and vesicular acetylcholine transporter (VAChT)—demonstrate significant degeneration that disrupts cortical neuromodulation, impairs attentional control, and diminishes adaptive plasticity [37]. Converging evidence suggests that dysfunctions in these excitatory and cholinergic systems may not only reflect downstream neurodegeneration but might also actively influence disease progression by destabilizing network homeostasis, amplifying inflammatory signaling, and probably facilitating the spread of pathological protein species [38,39,40].

In this study, we used multiplex immunofluorescence and 3D AI object identification and quantification approaches to examine and evaluate the distribution of TDP-43 inclusions in limbic system subfields and neuronal subtypes in a small group of AD and PART cases exhibiting distinct co-pathologies in a cross-sectional manner. Quantification of morphological colocalization and other pathological features could leverage 3D AI segmentation to derive complex metrics that define the structural quality and phenotypic complexity of TDP-43 aggregates. We predicted that TDP-43 would coincide with noradrenergic marker DβH, to be linked to modulatory neurons, rather than excitatory ones.

## 2. Methods and Protocols

### 2.1. Patient Recruitment

From December 2020 through October 2023, we assembled a deeply phenotyped cohort consisting of control individuals and cases representing PART-TDP, hAD-TDP, and hAD-TDP-LBD. Participants were evaluated either through Mayo Clinic’s clinical programs or through NIH-supported initiatives such as the Neurodegenerative Research Group and the Alzheimer’s Disease Research Center in Rochester, Minnesota. Control cases were defined as individuals who died without evidence of neurodegenerative pathology at autopsy. All participants underwent standardized behavioral and neuropathologic assessment by trained specialists (KAJ and RRR). Written informed consent was obtained in accordance with Mayo Clinic Institutional Review Board approval (IRB# 14-004401, IRB# 712-98) and the ethical principles outlined in the Declaration of Helsinki.

### 2.2. Neuropsychological Evaluation

Each participant completed a structured neurological examination performed by a behavioral neurologist, followed by a neuropsychological battery administered under the supervision of a board-certified neuropsychologist (MMM). Cognitive status was characterized by using established clinical instruments, including the MMSE for global cognition and the 15-item Boston Naming Test for language. Executive abilities were assessed with Trail Making Tests A and B (Trail A/B), and functional decline was quantified using the CDR sum of boxes (CDRSUM). Behavioral symptoms were documented using standard clinical measures. APOE genotyping (ε4 allele status) was determined using previously validated protocols [2].

### 2.3. Multiplex Immunofluorescence Studies

Two formalin-fixed, paraffin-embedded (FFPE) human brain tissue blocks encompassing the amygdala and hippocampal formation regions of interest (ROIs) were selected for detailed analysis. Eight anatomically defined ROIs were examined: the basolateral amygdala (amygdala-BL), centromedial amygdala (amygdala-CM), cornu Ammonis-1 (CA1), CA2/3, CA4, dentate gyrus (DG), subiculum (SUB), and entorhinal cortex (ERC) (Supplementary Figure S1B). Sections were cut at 10 μm thickness to preserve cellular architecture across layers.

Tissue preparation followed a sequential workflow. Sections were permeabilized in Triton-X 100 (0.3% in phosphate-buffered saline (PBS), room temperature, 30 min) and subjected to heat-mediated antigen retrieval in sodium citrate buffer (10 mM, pH 6.0, 80 °C for 30 min). To reduce nonspecific human IgG signal, slides were incubated overnight at 4 °C with F(ab’)_2_ fragments (Jackson ImmunoResearch, West Grove, PA, USA, catalog # 709-006-149, 1:35 in PBS, overnight at 4 °C). After blocking with normal donkey serum (NDS) (2%) and bovine serum albumin (BSA) (0.1%), primary antibodies targeting pTDP-43 (pS409/410) (CosmoBio, Carlsbad, CA, USA, mouse monoclonal, catalog #TIP-PTD-M01, lot #11-9-G6, 1:400 [41] or Proteintech, Rosemont, IL, USA, rabbit polyclonal, catalog #22309-1-AP, 1:200 [42]), total TDP-43 (rabbit polyclonal, catalog #10782-2-AP, 1:200 [43]), N-terminal (rabbit polyclonal, catalog #80002-1-RR, 1:200 [44]) and C-terminal (rabbit polyclonal, catalog #12892-1-AP, 1:200 [45]) TDP-43 fragments and neuronal subtype markers (DβH (Thermo-Fisher, Waltham, MA, USA, goat polyclonal, catalog #PA5-142848, 1:400), TH (Synaptic Systems, Goettingen, Germany, Guinea pig polyclonal, catalog #213 104, 1:200), VGLUT1 (Synaptic Systems, Goettingen, Germany, Guinea pig monoclonal, catalog #135 318, 1:200), ChAT (Sigma-Aldrich, Burlington, MA, USA, Goat polyclonal, catalog #AB144P, 1:100)) were applied overnight at 4 °C. Fluorophore-conjugated secondary antibodies (Alexa Fluor 488 Donkey Anti-Mouse IgG (H + L), Jackson Immunoresearch, West Grove, PA, USA, 715-545-151, Alexa Fluor 568 Donkey Anti-Rabbit IgG (H + L), Jackson Immunoresearch, West Grove, PA, USA, 711-575-152, DyLight™ 405 AffiniPure™ Donkey Anti-Guinea Pig IgG (H + L), Jackson Immunoresearch, West Grove, PA, USA, 706-475-148, Alexa Fluor^®^ 647 AffiniPure™ Donkey Anti-Guinea Pig IgG (H + L), Jackson Immunoresearch, West Grove, PA, USA, 706-605-148), DyLight™ 405 AffiniPure™ Donkey Anti-Rat IgG (H + L), Jackson Immunoresearch, West Grove, PA, USA, 712-475-153, Alexa Fluor^®^ 647 AffiniPure™ Donkey Anti-Chicken IgY (IgG) (H + L), Jackson Immunoresearch, West Grove, PA, USA, 703-605-155, Alexa Fluor^®^ 647 AffiniPure™ Donkey Anti-Goat IgG (H + L), Jackson Immunoresearch, West Grove, PA, USA, 705-605-147) were added the following day. Lipofuscin autofluorescence was quenched using lipofuscin autofluorescence quencher TrueBlack, (Biotium, Fremont, CA, USA, catalog #23007, three minutes, room temperature), and sections were mounted in antifade medium (Vector laboratories, Newark, CA, USA, VECTASHIELD Vibrance^®^ Antifade Mounting Medium (H-1700)). Omission controls confirmed specificity of labeling.

### 2.4. 3D AI-Assisted Object Recognition, Reconstruction and Relation Count Quantification

Multiplexed images were acquired using a Zeiss 980 LSM spinning-disk confocal (Oberkochen, Baden-Württemberg, Germany) system under uniform illumination settings. Image acquisition was performed blinded to diagnostic group. For each region, multiple fields (three to four) were sampled at 20× magnification, with spacing of at least 100 μm to avoid oversampling. Z-stacks consisted of ten optical planes (1 μm steps). We used a three-dimensional (3D) AI-based workflow implemented in AIVIA 15.0 (Leica Microsystems, Wetzlar, Germany) (Supplementary Figure S1A) [46,47] to quantify pTDP-43 inclusions and their spatial relationship to neuronal subtypes (Supplementary Figure S1B,C). Manual counting of the pTDP-43-neuronal-subtype colocalized particles in a representative amygdala-BL region is shown in Supplementary Figure S2E–H as the internal validation of this AI segmentation pipeline. Also, our method has been validated in prior studies [46,47]. Pixel classifiers were trained on four–five representative images from two–three cases per diagnostic group, with manual delineation of signal and background. In our workflow, independently sampled ROIs from the same cases were randomly allocated either to AI segmentation training or to downstream morphometric and colocalization measurements. Once trained, classifiers were incorporated into a custom analysis pipeline that reconstructed objects, identified labeled 3D structures, extracted morphometric features, and computed dual-marker colocalization. Specifically, the workflow identifies pTDP-43-positive and neuronal-subtype-specific objects in 3D space and evaluates their spatial relationship based on object-level overlap within segmented somatic and neuritic volumes, rather than proximity alone. In other words, only overlapping particles were considered colocalized or exhibited a relation count. The software also implemented parameters such as centroid-based distance thresholds and explicit touching-versus-overlap criteria. *Z*-axis overlap was handled through 3D object reconstruction rather than slice-by-slice proximity measures. All the stained particles were designated as positive without any subcategorization such as nuclear, cytoplasmic, soma, or neurite categorization or whether they were spherical or elongated. The software analyzed colocalization when pTDP-43- and neuronal-subtype-marker-labeled particles had at least one contact with each other in 3D space, which means that particles should overlap to be considered colocalized or in relation. In this regard, one particle can have no to multiple overlaps due to the size and localization. All raw czi files were processed through this supervised workflow and exported for downstream analysis.

### 2.5. Statistical Analysis

For all analyses, the biological unit was the individual case/participants (but not the slide or ROI), denoted as “n”. Data are presented as medians with interquartile ranges (Q1–Q3). Group comparisons involving more than two diagnostic categories were performed using Kruskal–Wallis tests; pairwise comparisons used Mann–Whitney U tests. Associations between neuronal-subtype-specific pTDP-43 burden and clinical or pathological variables were evaluated using Spearman rank correlations and plotted as heatmaps. False Discovery Rate (FDR) correction was applied to *p*-values generated by Spearman correlations to control for multiple comparisons. Dimensionality reduction was performed using principal component analysis (PCA) to identify dominant axes of variance. This simplified dataset complexity and highlighted primary variance trends, with every principal component acting as an orthogonal linear combination of the original variables that maximized explained variance for visualization purposes. All variables were z-scored prior to PCA. Hierarchical clustering analysis (HCA) explored multiscale similarities by iteratively categorizing samples according to pairwise relatedness by the Ward method, creating a dendrogram that illustrated nested connections built from features combining brain regions, inclusions, and neuronal subtypes. We performed subject-level analyses adjusting for age as a covariate, as well as sensitivity analyses performed using age-matched subsets. All statistical procedures were conducted in GraphPad Prism 11 by investigators blinded to diagnostic status, and missing values for any PCA or HCA variable were excluded.

## 3. Results

### 3.1. Cohort Characteristics

Across all 15 cases, the median age at presentation was 93 years with an average age of 87.5 years. All the PART and hAD cases were at similar ages at death (93 ± 5.5), whereas controls were younger (67 ± 12.5), which we used to illustrate neuronal subtype labeling patterns under absent neurodegenerative conditions. Four patients (26.7%) were female. TDP-43 pathological stage was similar in each disease group, demonstrating low variability of distribution of the TDP-43 inclusions. Patient demographics and clinical test scores are outlined in Table 1 and Table 2 respectively.

### 3.2. pTDP-43 Inclusions in Noradrenergic, Dopaminergic, and Cholinergic Neurons and Glutamatergic Terminals of Amygdala

We employed a 3D AI object recognition, reconstruction, and analysis workflow in age-adjusted PART-TDP, hAD-TDP, and hAD-TDP-LBD groups to investigate TDP-43 inclusions, fragments and the spatial relationship between neuronal subtypes in each group; a schematic illustration of this workflow is shown in Supplementary Figure S1A. Controls served as a visual aid to compare neuronal subtype marker immunostaining differences but were not included in TDP-43 colocalization analyses due to a lack of pathology. Across the amygdala, ERC, and hippocampal subfields, pTDP-43 colocalization with DβH, Vglut1, TH, and ChAT revealed region- and pathology-specific patterns. 3D-reconstructed DβH-positive neurons and neurites together with orthogonal x and y axes in a representative CA1 region are demonstrated in Supplementary Figures S1B and C, respectively, to visualize how segmentation, reconstruction and colocalization pipelines are performed throughout the study.

**Figure 1:**
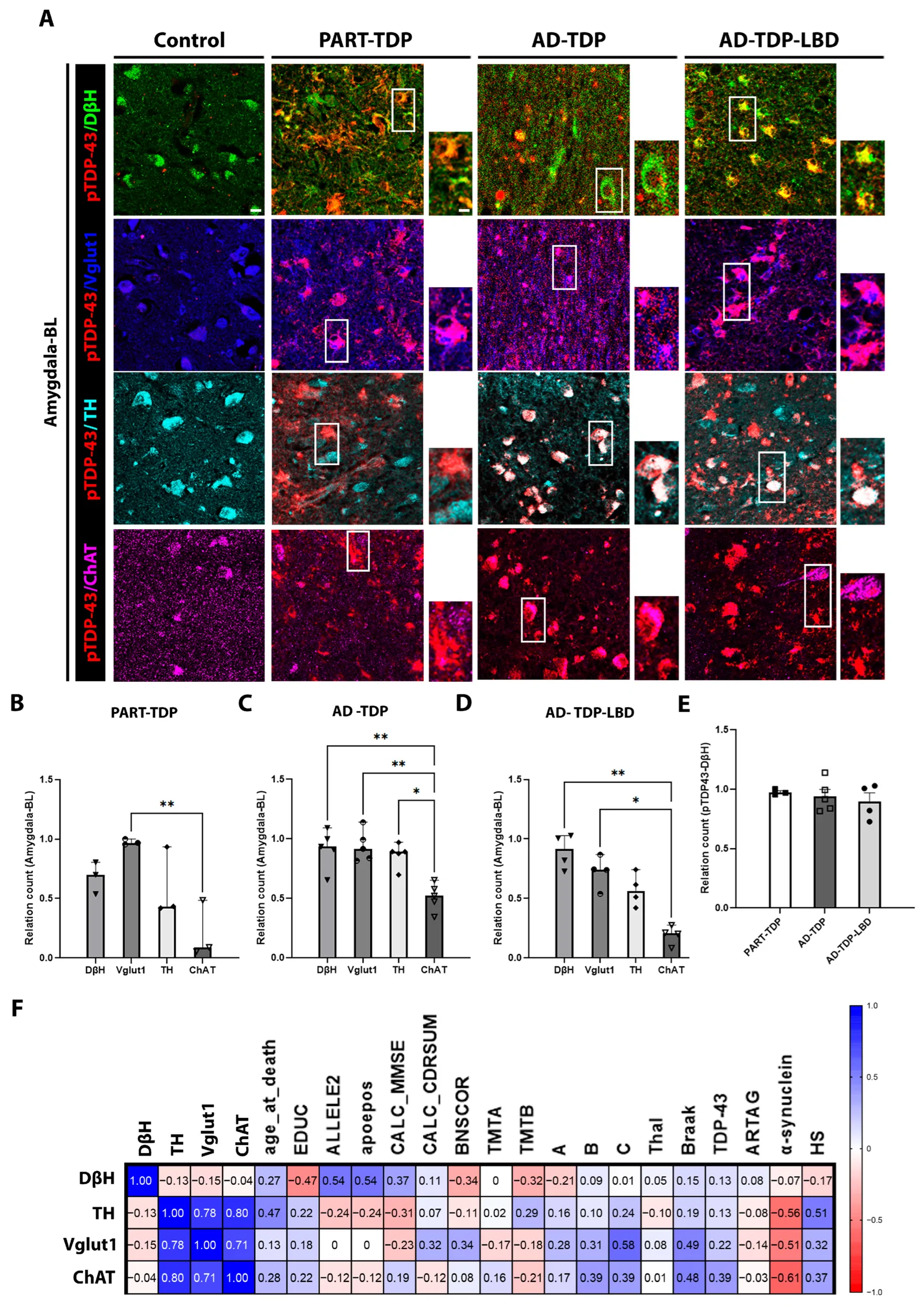
TDP-43 accumulation in noradrenergic, dopaminergic, and cholinergic neurons and glutamatergic terminals in amygdala-BL. (**A**) Immunofluorescent staining of the controls, PART-TDP, AD-TDP and AD-TDP-LBD with pTDP-43 (red), DβH (green), Vglut1 (blue), TH (cyan), and ChAT (magenta). Each row represents merged images of pTDP-43 and DβH, Vglut1, TH and ChAT to demonstrate spatial relationships of pTDP-43 aggregates in each condition. White rectangles show where the inserts are taken. Scale bars = 20 µm. AI segmentation and recognition of 3D objects from the confocal z-stack in each staining are performed. The relation counts between pTDP-43 and all markers are compared in (**B**) PART-TDP, (**C**) AD-TDP and (**D**) AD-TDP-LBD cases. (**E**) Relation count comparison of DβH (pTDP43–DβH) as the most differentiating marker in each group. (**F**) Representative Spearman correlation heatmap with respective coefficients between relation counts and clinicopathological assessments. Red and blue denote negative and positive correlations. * *p* < 0.05; ** *p* < 0.01. Kruskal–Wallis for grouped variables; Mann–Whitney U for pairwise comparisons.

In amygdala-BL, pTDP-43–DβH colocalization was greatest in hAD-TDP and hAD-TDP-LBD and lowest in PART-TDP, whereas pTDP-43–Vglut1 was highest in PART-TDP and pTDP-43–TH was highest in hAD-TDP (Figure 1A–E, Supplementary Figure S2A–D). pTDP-43–ChAT colocalization was lowest overall but relatively increased in hAD-TDP (Figure 1A–D, Supplementary Figure S2D). We also deployed manual counting in amygdala-BL together with our AI-based workflow for object recognition, reconstruction, and quantitative analysis, verifying that its performance closely matched manual quantification (Supplementary Figure S2E–H).

DβH correlated positively with APOE and negatively with education (Figure 1F). Vglut1 correlated positively with TH, ChAT, neuritic plaque score (C), and Braak stage and negatively with LBD. TH was positively correlated with Vglut1, ChAT, HS, and age at death, and negatively correlated with LBD. ChAT correlated positively with Vglut1, TH, and Braak stage and negatively with LBD.

**Figure 2:**
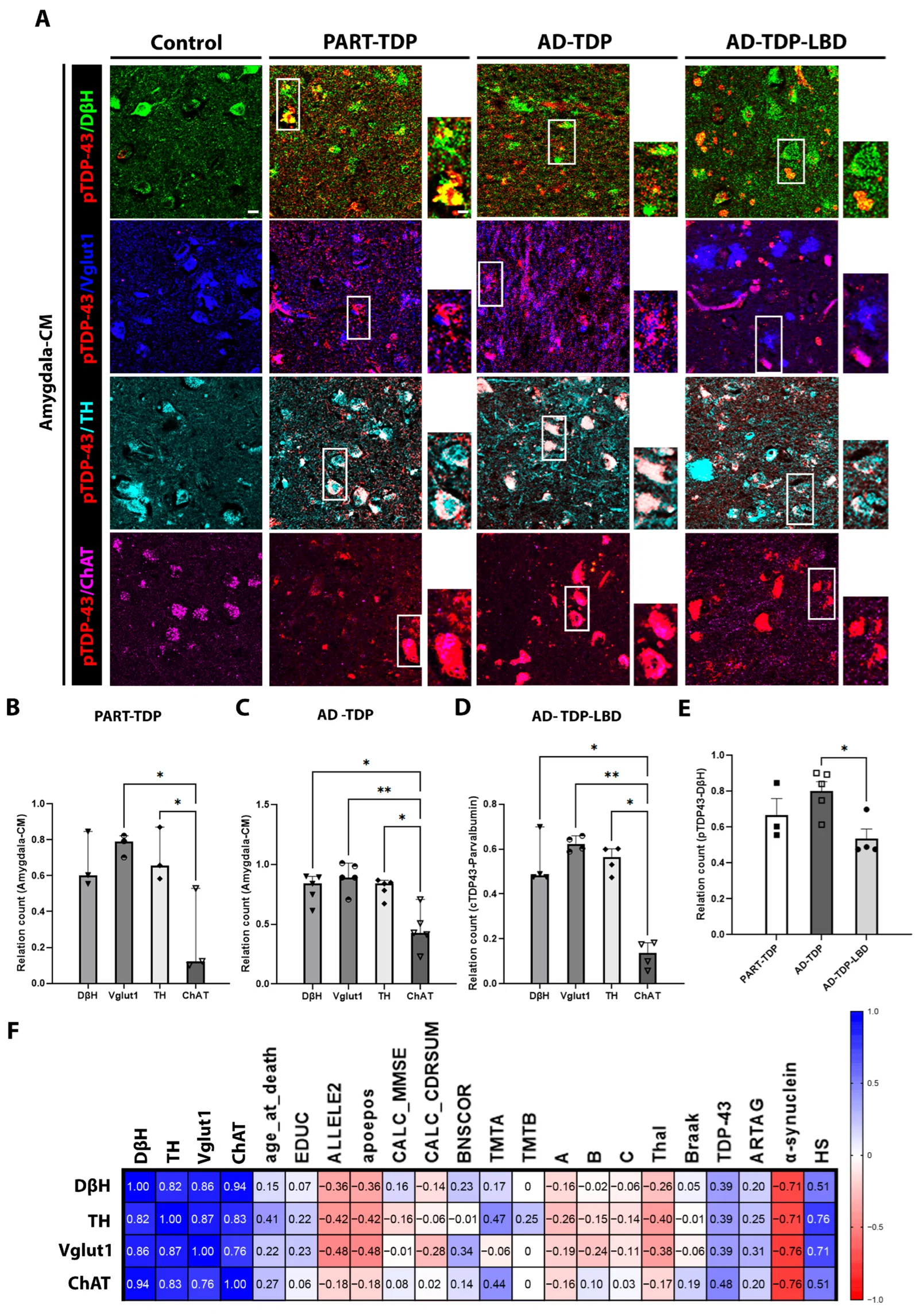
TDP-43 accumulation in noradrenergic, dopaminergic, and cholinergic neurons and glutamatergic terminals in amygdala-CM. (**A**) Immunofluorescent staining of the controls, PART-TDP, AD-TDP and AD-TDP-LBD with pTDP-43 (red), DβH (green), Vglut1 (blue), TH (cyan), and ChAT (magenta). Each row represents merged images of pTDP-43 and DβH, Vglut1, TH and ChAT to demonstrate spatial relationships of pTDP-43 aggregates in each condition. White rectangles show where the inserts are taken. Scale bars = 20 µm. AI segmentation and recognition of 3D objects from the confocal z-stack in each staining are performed. The relation counts between pTDP-43 and all markers are compared in (**B**) PART-TDP, (**C**) AD-TDP and (**D**) AD-TDP-LBD cases. (**E**) Relation count comparison of DβH (pTDP43–DβH) as the most differentiating marker in each group. (**F**) Representative Spearman correlation heatmap with respective coefficients between relation counts and clinicopathological assessments. Red and blue denote negative and positive correlations. * *p* < 0.05; ** *p* < 0.01. Kruskal–Wallis for grouped variables; Mann–Whitney U for pairwise comparisons.

In amygdala-CM, DβH colocalization was greatest in hAD-TDP and lowest in hAD-TDP-LBD, whereas Vglut1 was highest in PART-TDP and TH in hAD-TDP (Figure 2A–E, Supplementary Figure S2I–L). ChAT colocalization remained lowest overall but relatively enriched in hAD-TDP (Figure 2A–D, Supplementary Figure S2L). To determine whether neurodegeneration has an impact on relation count analyses, we quantified DβH-positive neurons in both amygdala regions (Supplementary Figure S3A,B) and normalized relation counts to neurons in each respective ROI (Supplementary Figure S3C–J). The AD-TDP-LBD group displayed a smaller number of neurons than PART-TDP in amygdala-BL (Supplementary Figure S3A), whereas the AD-TDP group showed the same pattern in amygdala-CM (Supplementary Figure S3B). TDP-43 relation counts normalized to total number of DβH-labeled neurons showed similar patterns across the markers in both amygdala regions compared to non-normalized counterparts (Supplementary Figure S3C–J).

DβH, Vglut1, TH, and ChAT were positively intercorrelated and associated with HS, and each showed negative associations with LBD; Vglut1 was additionally negatively correlated with APOE (Figure 2F).

### 3.3. pTDP-43 Inclusions in Noradrenergic, Dopaminergic, and Cholinergic Neurons and Glutamatergic Terminals of Hippocampal Subfields

**Figure 3:**
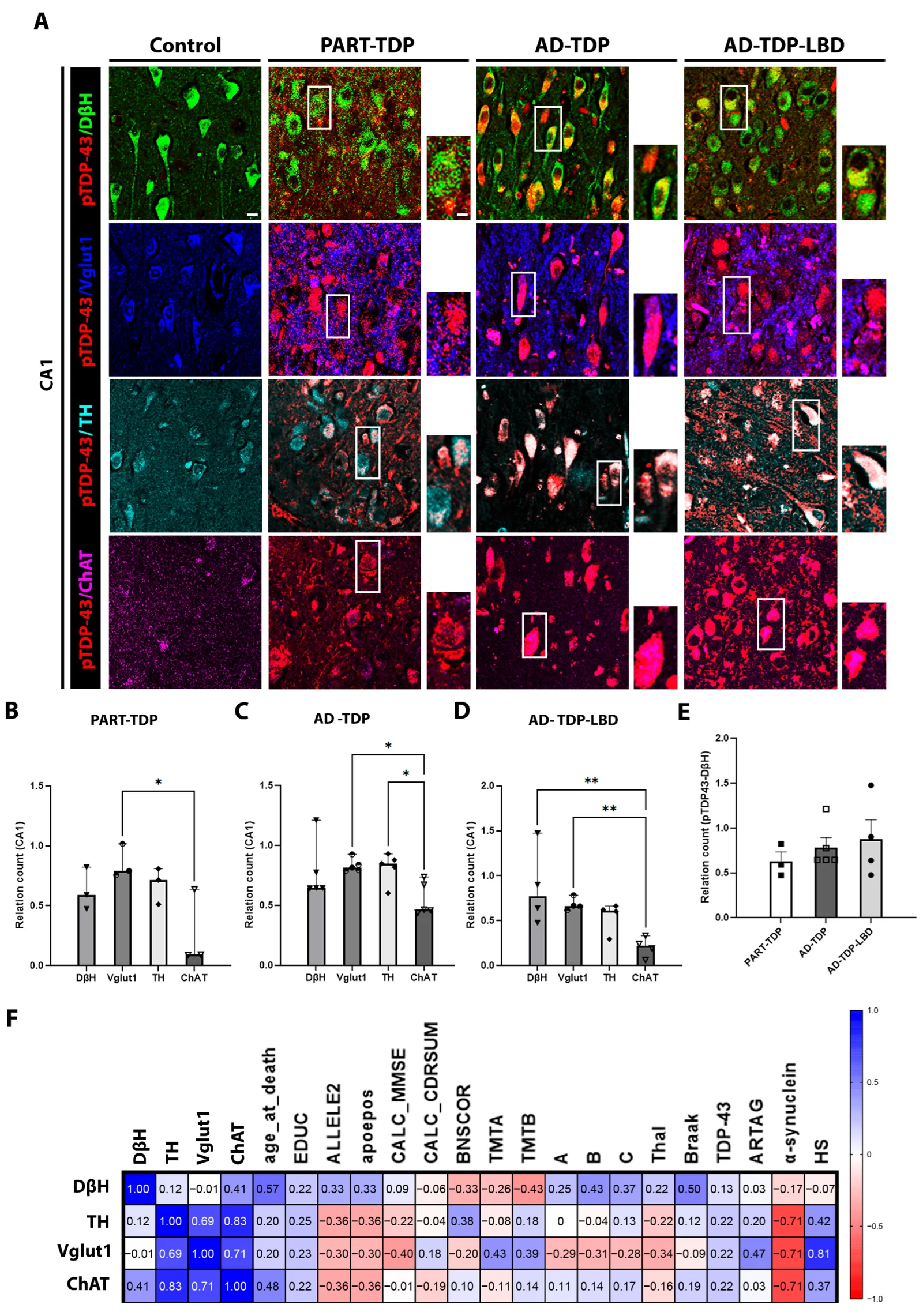
TDP-43 accumulation in noradrenergic, dopaminergic, and cholinergic neurons and glutamatergic terminals in CA1. (**A**) Immunofluorescent staining of the controls, PART-TDP, AD-TDP and AD-TDP-LBD with pTDP-43 (red), DβH (green), Vglut1 (blue), TH (cyan), and ChAT (magenta). Each row represents merged images of pTDP-43 and DβH, Vglut1, TH and ChAT to demonstrate spatial relationships of pTDP-43 aggregates in each condition. Scale bars = 20 µm. AI segmentation and recognition of 3D objects from the confocal z-stack in each staining are performed. The relation counts between pTDP-43 and all markers are compared in (**B**) PART-TDP, (**C**) AD-TDP and (**D**) AD-TDP-LBD cases. (**E**) Relation count comparison of DβH (pTDP43–DβH) as the most differentiating marker in each group. (**F**) Representative Spearman correlation heatmap with respective coefficients between relation counts and clinicopathological assessments. Red and blue denote negative and positive correlations. * *p* < 0.05; ** *p* < 0.01. Kruskal–Wallis for grouped variables; Mann–Whitney U for pairwise comparisons.

Across hippocampal subfields, neuronal-subtype-specific pTDP-43 colocalizations showed convergent yet regionally defining associations with neuropathologic and clinical measures. DβH colocalization was generally greatest in hAD-TDP (with CA1 enriched in hAD-TDP-LBD and lowest levels frequently in PART-TDP or hAD-TDP-LBD) (Figure 3A–E, Supplementary Figure S3A–D). It was positively associated with age at death, Braak stage, APOE genotype, amyloid measures (A–C), and, in several regions, TH and ChAT. Negative associations were observed with cognitive measures (Trail A/B), BNT, and LBD in select subfields (Supplementary Figure S3F).

**Figure 4:**
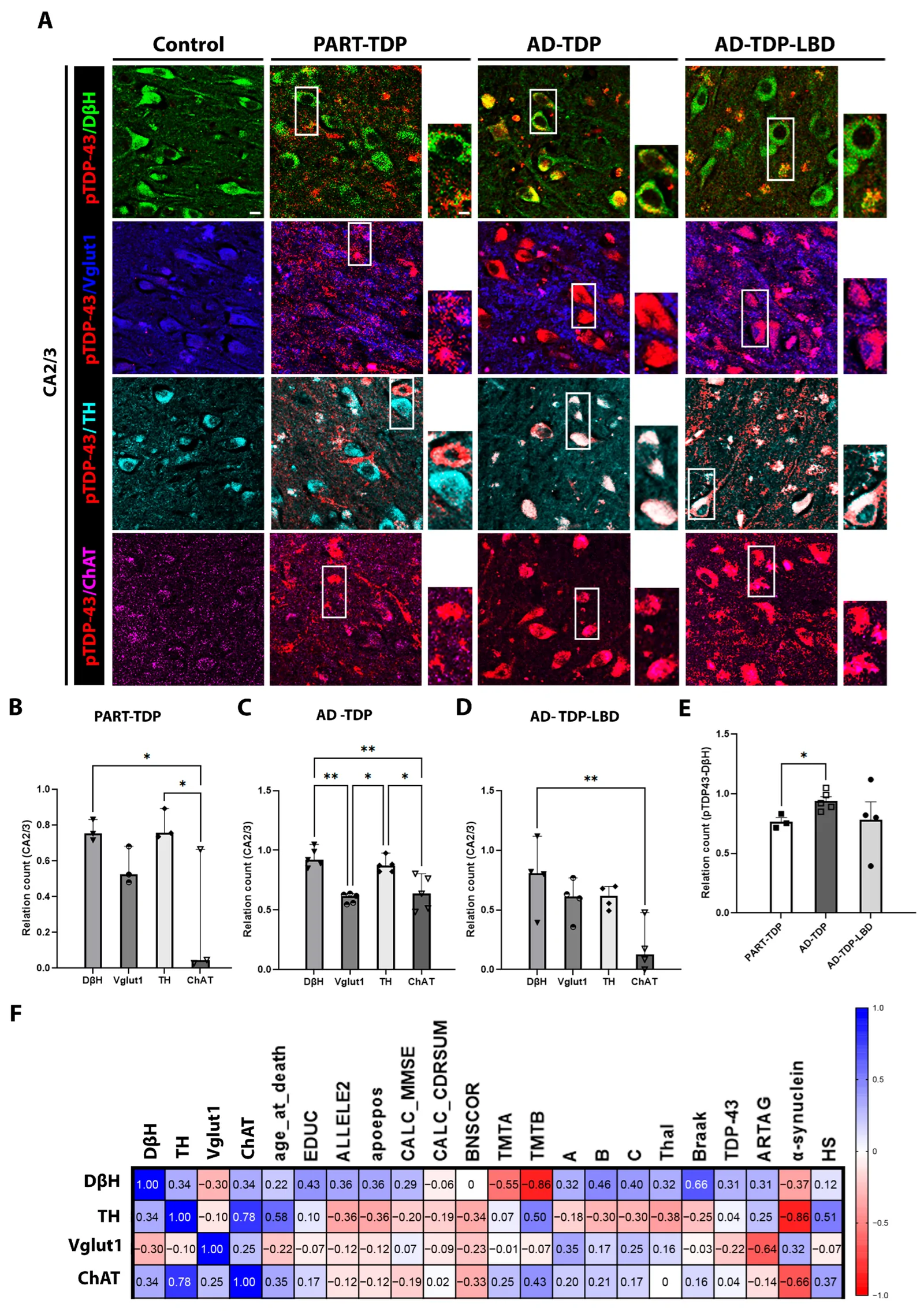
TDP-43 accumulation in noradrenergic, dopaminergic, and cholinergic neurons and glutamatergic terminals in CA2/3. (**A**) Immunofluorescent staining of the controls, PART-TDP, AD-TDP and AD-TDP-LBD with pTDP-43 (red), DβH (green), Vglut1 (blue), TH (cyan), and ChAT (magenta). Each row represents merged images of pTDP-43 and DβH, Vglut1, TH and ChAT to demonstrate spatial relationships of pTDP-43 aggregates in each condition. White rectangles show where the inserts are taken. Scale bars = 20 µm. AI segmentation and recognition of 3D objects from the confocal z-stack in each staining are performed. The relation counts between pTDP-43 and all markers are compared in (**B**) PART-TDP, (**C**) AD-TDP and (**D**) AD-TDP-LBD cases. (**E**) Relation count comparison of DβH (pTDP43- DβH) as the most differentiating marker in each group. (**F**) Representative Spearman correlation heatmap with respective coefficients between relation counts and clinicopathological assessments. Red and blue denote negative and positive correlations. * *p* < 0.05; ** *p* < 0.01. Kruskal–Wallis for grouped variables; Mann–Whitney U for pairwise comparisons.

Vglut1 colocalization showed minimal group differences in CA1–CA4 and SUB (Figure 3 and Figure 4 and Supplementary Figures S4 and S5) but was increased in PART-TDP in the DG (Supplementary Figure S4A–D). Across subfields, Vglut1 positively correlated with TH, ChAT, HS, and, region-specifically, Trail A/B performance, while showing negative associations with LBD, ARTAG, education, BNT, MMSE, and neuritic plaque score (C) (Figure 3 and Figure 4F, Supplementary Figures S4 and S5F).

TH and ChAT colocalizations were consistently highest in hAD-TDP (with region-specific reductions in hAD-TDP-LBD or PART-TDP) (Figure 3 and Figure 4 and Supplementary Figures S4–S6). TH and ChAT were strongly intercorrelated across all subfields. Both markers were positively associated with age at death and HS and negatively associated with LBD. Additional region-specific relationships included inverse correlations between TH and CDR sum of boxes (subiculum) and between ChAT and LBD across nearly all hippocampal regions (Figure 3 and Figure 4F, Supplementary Figures S4 and S5F).

Collectively, hippocampal subfields demonstrated coordinated neuronal subtype associations with amyloid, tau staging, APOE genotype, and cognitive performance, with LBD again emerging with a consistent negative correlation across noradrenergic systems.

### 3.4. pTDP-43 Inclusions in Noradrenergic, Dopaminergic, and Cholinergic Neurons and Glutamatergic Terminals of Entorhinal Cortex

**Figure 5:**
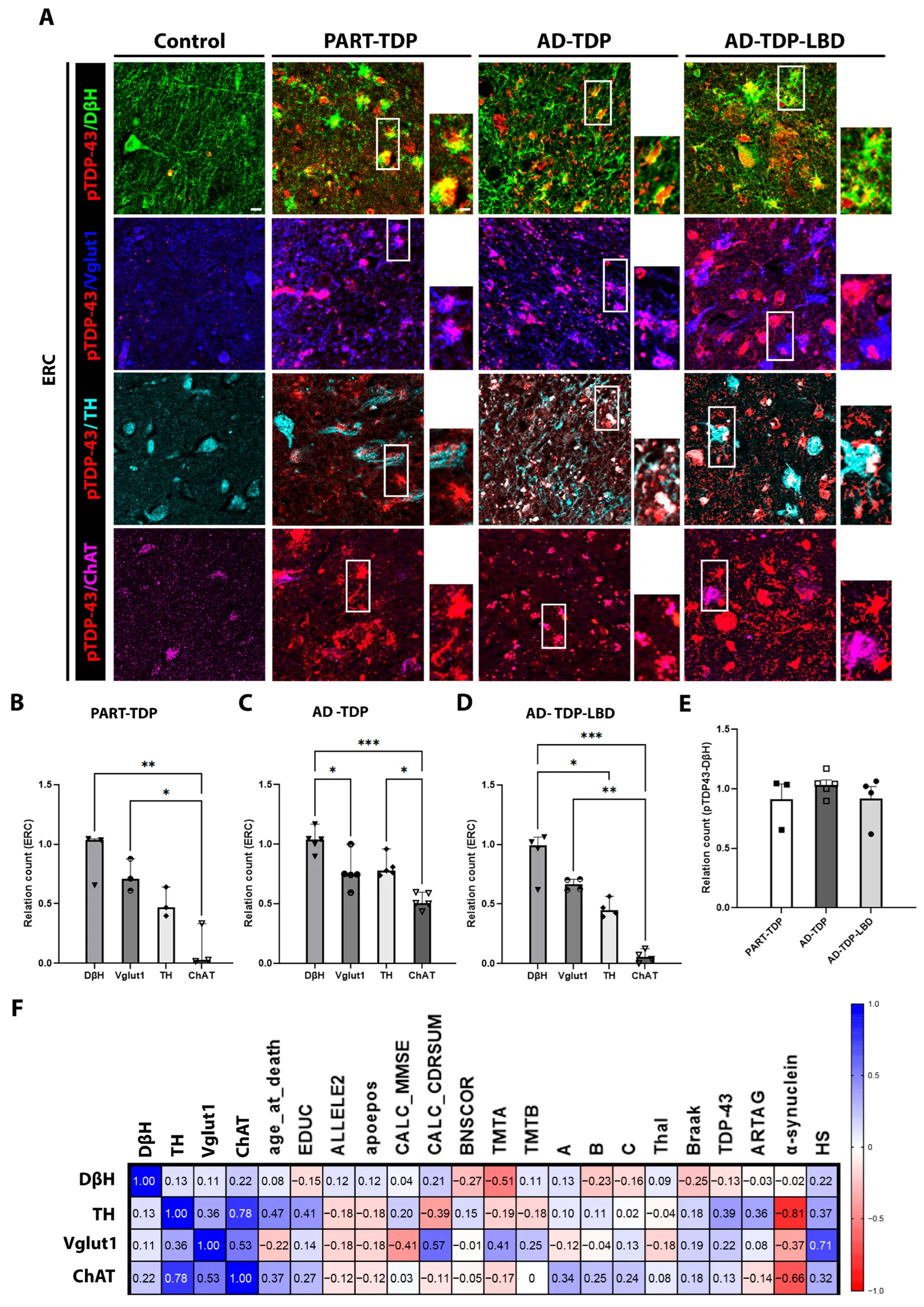
TDP-43 accumulation in noradrenergic, dopaminergic, and cholinergic neurons and glutamatergic terminals in ERC. (**A**) Immunofluorescent staining of the controls, PART-TDP, AD-TDP and AD-TDP-LBD with pTDP-43 (red), DβH (green), Vglut1 (blue), TH (cyan), and ChAT (magenta). Each row represents merged images of pTDP-43 and DβH, Vglut1, TH and ChAT to demonstrate spatial relationships of pTDP-43 aggregates in each condition. White rectangles show where the inserts are taken. Scale bars = 20 µm. AI segmentation and recognition of 3D objects from the confocal z-stack in each staining are performed. The relation counts between pTDP-43 and all markers are compared in (**B**) PART-TDP, (**C**) AD-TDP and (**D**) AD-TDP-LBD cases. (**E**) Relation count comparison of DβH (pTDP43- DβH) as the most differentiating marker in each group. (**F**) Representative Spearman correlation heatmap with respective coefficients between relation counts and clinicopathological assessments. Red and blue denote negative and positive correlations. * *p* < 0.05; ** *p* < 0.01; *** *p* < 0.001. Kruskal–Wallis for grouped variables; Mann–Whitney U for pairwise comparisons.

pTDP-43 colocalization with neurotransmitter-specific markers revealed pathology- and region-dependent patterns in ERC. DβH-colocalized particles were greatest in hAD-TDP and lowest in hAD-TDP-LBD, whereas Vglut1 colocalization was highest in PART-TDP (Figure 5A–E). TH colocalization was greatest in hAD-TDP, and ChAT colocalization was lowest overall, with relative enrichment in hAD-TDP (Figure 5A–D). Spearman correlation analyses indicated that DβH negatively correlated with Trail A performance. Vglut1 positively correlated with ChAT and hippocampal sclerosis (HS). TH correlated positively with ChAT and age at death and negatively with LBD. ChAT correlated positively with TH and HS and negatively with LBD (Figure 5F). These findings indicate the coordinated changes across noradrenergic, dopaminergic, and cholinergic neurons and glutamatergic terminals. Also, LBD and cognitive measures emerged as key factors associated with pTDP-43, coinciding with neuromodulatory neurons.

### 3.5. Multiregional pTDP-43 and Pathology-Based Principal Component and Hierarchical Clustering Analysis

**Figure 6:**
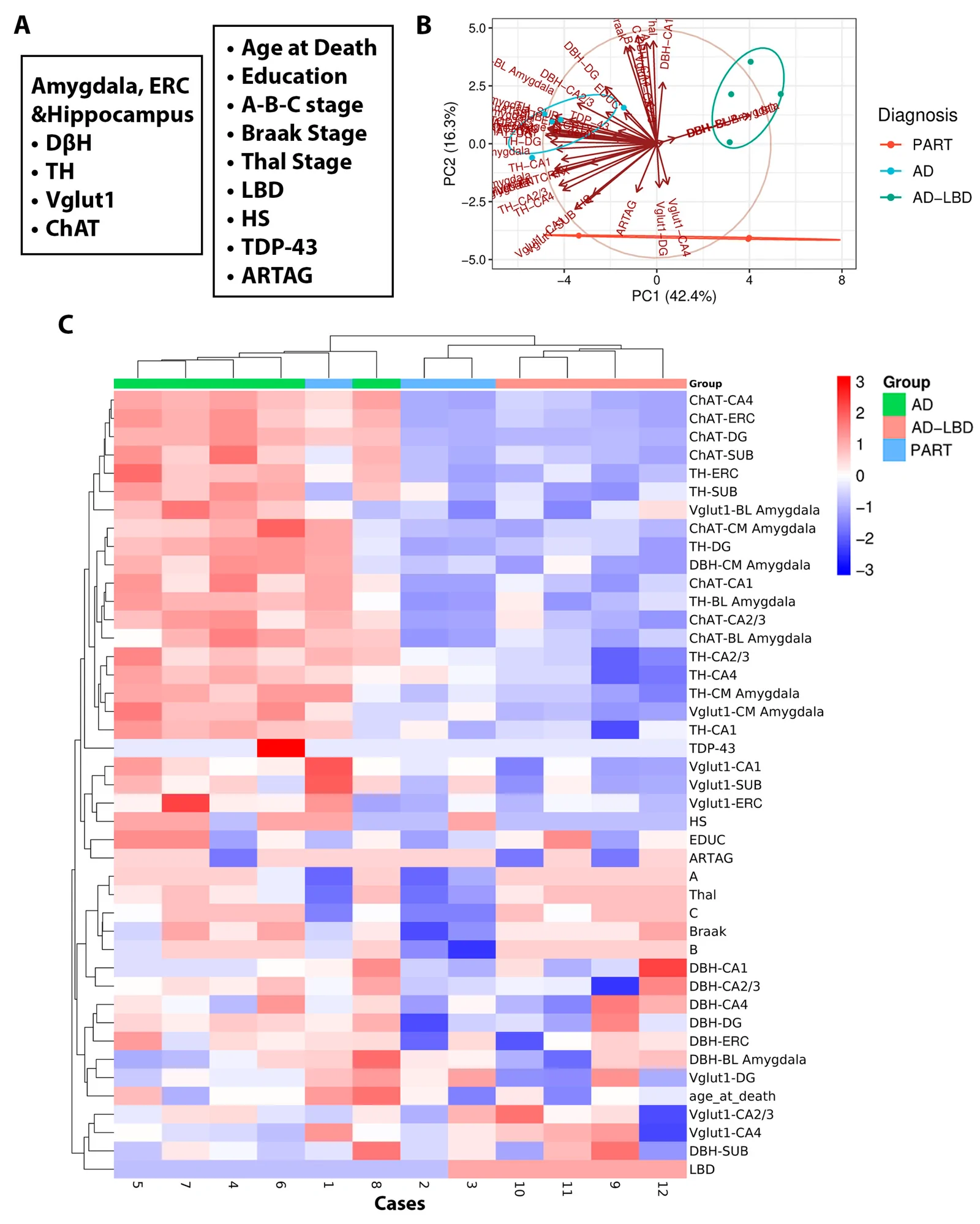
Principal component (PCA) and hierarchical cluster analyses (HCA) of clinicopathological assessments with all pTDP-43 colocalization data. (**A**) Schematic of parameters of interest. (**B**) PCA of the three distinct groups, PART-TDP, AD-TDP, and AD-TDP-LBD. n = 3–5 cases/group. Arrows indicate directionality of each parameter. Ellipsoids harbor cases belonging to the same neuropathological diagnostic group. The PCA circle shows the correlation via maximal possible variance. (**C**) Hierarchical cluster analysis with Euclidean Ward method of 12 cases. Dendrograms showing the proximity of each case and group. Green: AD-TDP; orange: AD-TDP-LBD; blue: PART-TDP. Z scores between −3 and 3 are shown as a blue-to-red spectrum.

**Figure 7:**
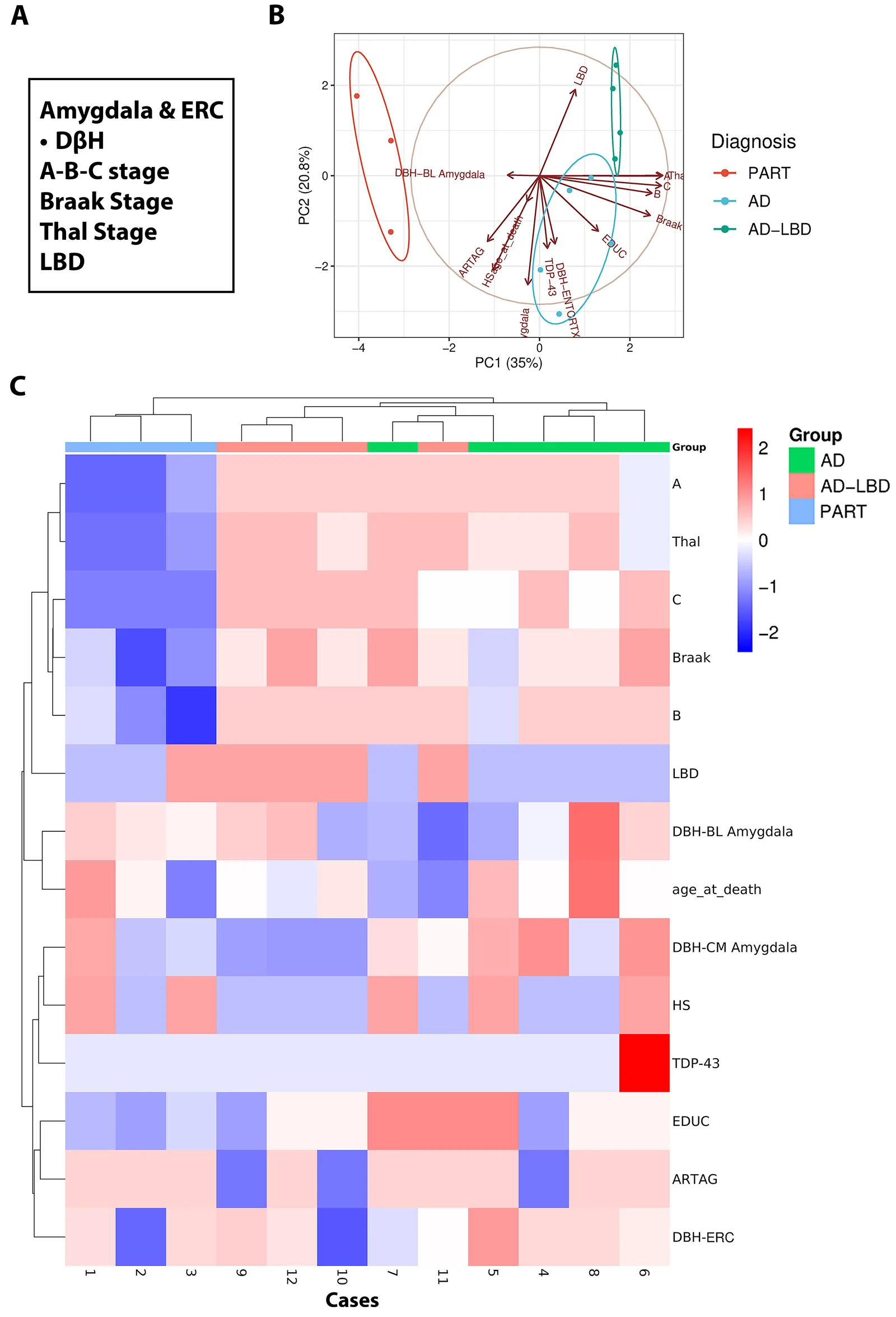
PCA and HCA using clinicopathological assessments with pTDP-43 and DβH colocalization data in amygdala and ERC. (**A**) Schematic of parameters of interest. (**B**) PCA of the three distinct groups, PART-TDP, AD-TDP, and AD-TDP-LBD. n = 3–5 cases/group. Arrows indicate directionality of each parameter. Ellipsoids harbor cases belonging to the same neuropathological diagnostic group. The PCA circle shows the correlation via maximal possible variance. (**C**) HCA with Euclidean Ward method of 12 cases. Dendrograms showing the proximity of each case and group. Green: AD-TDP; orange: AD-TDP-LBD; blue: PART-TDP. Z scores between −2 and 2 are shown as a blue-to-red spectrum.

Principal component analysis (PCA) and hierarchical clustering (HCA) of multiregional data in this 12-case cross-sectional cohort robustly separated hAD-TDP, hAD-TDP-LBD, and PART-TDP groups (Figure 6 and Figure 7). LBD was again demonstrated as a major contributor to variance and cluster structure (Figure 6 and Figure 7B). Extended analyses encompassing the hippocampus and combined amygdala–hippocampus–ERC datasets similarly differentiated groups, although partial overlap between PART-TDP and hAD-TDP was observed in cases with lower amyloid burden (Figure 6 and Figure 7). Hierarchical clustering revealed subgroup shifts consistent with amyloid load, with individual cases of PART-TDP with low amyloid occasionally clustering within hAD-TDP, and one hAD-TDP case clustering closer to hAD-TDP-LBD (Figure 6B,C). In amygdala-focused models incorporating DβH and neuropathologic staging metrics (A, B, C, Thal phase, Braak stage, LBD), pathological groups were perfectly segregated (Figure 7B). Across regions, colocalization patterns of DβH provided the strongest discrimination among pathological groups. We also employed neuropathological data only (Supplementary Figure S9) and pTDP-43 colocalization count-centered analyses (Supplementary Figure S10). Neuropathological assessments such as Braak, Thal and CERAD staging were able to distinguish PART from AD counterparts by amyloid presence and tau staging, whereas this approach failed to differentiate AD-TDP subgroups (Supplementary Figure S9C). On the other hand, pTDP-43-neuronal-subtype colocalization counts in amygdala and hippocampal subfields separated AD-TDP subgroups, but PART could not be distinguished from AD counterparts without amyloid and tau staging information (Supplementary Figure S10C). Overall, these analyses demonstrate that multi-marker pTDP-43 colocalization combined with neuropathologic staging metrics reliably distinguishes AD subtypes and underscores the influential role of LBD and amyloid burden in defining cluster structure. These findings may provide a framework for integrating cellular vulnerability, neuronal subtype identity, and neuropathologic staging to classify and differentiate AD-related TDP-43 proteinopathies across the amygdala, hippocampus, and ERC.

## 4. Discussion

We demonstrated that limbic TDP-43 pathology is associated with noradrenergic and dopaminergic neuronal populations with additional but more limited involvement of pure glutamatergic terminals and cholinergic cells to a comparable extent among PART, hAD-TDP, and hAD-TDP-LBD groups. This TDP-43 accumulation pattern was consistent across the amygdala, hippocampal subfields, and ERC, whereas it was further modulated by the presence of LBD, which emerged as a major determinant of regional and dopaminergic neuronal vulnerability [48] assessed by subject-level analyses adjusting for age as a covariate as well. We also report the outcomes of these additional analyses and clarify that the principal findings remained unchanged after accounting for age, supporting the robustness of the observed associations. Together, these results reveal that the cellular landscape with which TDP-43 coincides may contribute to modulatory and excitatory circuits as key drivers of limbic dysfunction in the AD-PART neurodegenerative spectrum [13,49,50,51].

### 4.1. Noradrenergic Neurons as a Major Site of TDP-43 Accumulation

A second major finding is the prominent involvement of TDP-43 in noradrenergic neurons, marked by DβH and TH [28,31,52]. Across nearly all regions, DβH-positive neurons showed high pTDP-43 colocalization, particularly in hAD-TDP and hAD-TDP-LBD. Although there are slight differences in the total number of DβH-labeled neurons, overall relation count analyses were not influenced by these changes due to relatively homogeneous neurodegeneration across groups. Moreover, the labeling pattern and shape denominators of DβH also demonstrated that these subtypes of neurons closely resemble tangle-associated TDP-43 (TATs) [47], and yet we should cautiously define them as spatial associations between pTDP-43 and DβH-positive neurons. Our group has previously demonstrated the role of these TATs in the AD-FTLD spectrum [53]. This pattern is notable given the early degeneration of the locus coeruleus in AD, where noradrenergic signaling plays essential roles in arousal, attention initiation, and memory consolidation [31,48,54]. The strong correlations among TDP-43 and DβH colocalizations with *APOE* genotype, amyloid burden, and tau staging in this small cohort suggest that noradrenergic neurons may represent a central convergence for AD-related co-pathologies, with TDP-43 acting as a potential molecular insult based on prior work [29,31,54]; however, we also need to note that further studies are warranted due to the exploratory nature of our small cohort.

The observation that TH-positive neurons showed the highest pTDP-43 burden in hAD-TDP but the lowest in hAD-TDP-LBD may further emphasize the TDP and LBD interaction [55]. Given that TH-expressing neurons in the amygdala and hippocampus include both dopaminergic and noradrenergic subtypes, these findings may correlate with the fact that LBD has a negative impact on dopaminergic neuron survival and vulnerability [16,51,56], potentially through competition for proteostatic machinery or altered axonal transport driven by dysfunctional TDP-43 [48,57], but this link needs to be further investigated.

### 4.2. Involvement of TDP-43 in Glutamatergic Terminals and Cholinergic Neurons

Along with noradrenergic neurons, despite being abundant and structurally vulnerable in AD [34,36,58], Vglut1-labeled glutamatergic terminals also showed significant pTDP-43 colocalization especially at the neurite level. However, these neurons are not purely glutamatergic and yet they also harbor the noradrenergic/dopaminergic group due to colocalization patterns [59]. On the other hand, the few Vglut1-only-positive colocalizations observed were restricted to PART-TDP and likely reflect preserved neuronal viability [36]. This relative sparing might also be associated with less glutamatergic terminal degeneration in PART compared to AD. These findings may contribute to distinctive evolution of TDP-43 pathology in AD and PART, which warrants stage-focused assessment in the future. We also want to note that TDP-43 might preferentially target modulatory circuits rather than principal excitatory neurons, distinguishing it from tau-mediated neurodegeneration [60], although we need to cautiously raise this possibility due to extensive neurodegeneration in these cases and a lack of early-stage participants in this cohort.

Cholinergic neurons were sparse across all regions, consistent with their known anatomical distribution [38,39], though few detectable ChAT-positive neurons frequently colocalized with pTDP-43 in hAD-TDP. Although limited in number, this finding aligns with the well-established cholinergic deficits in AD [61] and it suggests that TDP-43 may contribute to cholinergic dysfunction in a subset of cases, particularly those without LBD [40].

### 4.3. Lewy Body Disease as a Major Modifier of TDP-43 Cellular Distribution

Across all analyses, our clustering approaches consistently revealed that TDP 43-associated diagnostic groups form biologically meaningful separations. LBD emerged as a negative correlate of pTDP-43 colocalization, particularly in DβH and TH subtypes. HCA and PCA further demonstrated that amyloid burden and LBD status were primary drivers of group separation, often overriding tau burden due to similar Braak staging across the cases. These results indicate that synucleinopathy may alter the cellular landscape of TDP-43 pathology along with region- and neuron-selective vulnerability [16,40,51,56,62]. The most striking data across multiple grouping variables came from the amygdala marker panel (DβH) combined with A-B-C, Thal, Braak, and LBD staging, which produced the clearest group separation of any dataset. PCA perfectly separated PART-TDP, hAD-TDP, and hAD-TDP-LBD, indicating that cell-type-specific TDP 43 colocalization patterns in the amygdala may carry a diagnostic signal. HCA further reinforced this structure, showing that PART-TDP cases cluster closer to hAD-TDP-LBD when amyloid burden is low, highlighting amyloid as a key grouping modifier. On the other hand, one PART case with low amyloid levels clustered within the hAD-TDP group, which illustrates how amyloid levels can override TDP 43-based grouping in borderline cases. Running the analyses with neuropathological staging metrics (Braak, Thal, CERAD) distinguished PART from AD, reflecting their sensitivity to amyloid deposition and tau progression as well. However, these global indices were unable to separate AD-TDP cases with and without LBD pathology. In contrast, pTDP-43 colocalization counts across neuronal subtypes in the amygdala and hippocampal subfields differentiated AD-TDP subgroups, indicating that cell-type-specific TDP-43 burden may capture heterogeneity missed by traditional staging. Yet this approach alone could not distinguish PART from AD without amyloid and tau information, underscoring that these methods provide complementary but non-overlapping axes of disease classification in this exploratory small cohort. Lastly, the reduced pTDP-43 burden in several neuronal subtypes in hAD-TDP-LBD may reflect either competitive sequestration of aggregation-prone proteins or accelerated further neuronal loss as quantified in this study, both of which warrant further mechanistic investigation [51,62].

### 4.4. Functional Implications for Limbic Circuit Dysfunction

The convergence of TDP-43 pathology on noradrenergic neurons may provide a mechanistic framework for understanding the associated limbic dysfunction while noradrenergic projections modulate arousal, attention, and memory encoding [28]. Disruption of these systems might be sufficient to produce network hyperexcitability and emotional dysregulation, which can occur in AD and LATE [48,52,63,64,65]. The strong correlations between pTDP-43 colocalization in our cohort and cognitive measures in the hippocampus but not the amygdala—including Trail A/B, BNT, MMSE, and CDR—together with previous studies support the idea that cell-type-specific TDP-43 pathology may contribute to clinical impairment, rather than serving as an epiphenomenon of advanced disease [2,13,23,49]. It should also be noted that these exploratory results suggest a correlative relationship rather than a mechanistic or causal inference until further translational research is conducted.

### 4.5. Limitations and Future Directions

This study is limited by its modest sample size, significantly younger control cases, and the inherent variability of human postmortem tissue, which makes it exploratory in nature. We acknowledge that age-related TDP-43 accumulation may create a confounding effect, and thus, we incorporated controls solely to illustrate the labeling patterns of multiple neuronal subtype markers under non-degenerative circumstances to provide a visual reference for the multiplex immunofluorescence analyses due to the unavailability of age-matched cases with absent neurodegenerative changes. We also had to scrutinize the selection of representative participants for each designated group, which might have introduced additional bias to the limited size, which also creates a major statistical limitation. Moreover, although it has been shown by different cohorts that TDP-43 exists in between 16 and 50% of LBD cases [11,12], AD-TDP-LBD cases might have more representation in our cohort; however, our study acts as a proof of concept of how TDP-43 inclusions are distributed throughout the AD spectrum. Also, the cohort in which we assess TDP-43 in AD comprises much older cases due to the disease course, though this is a known biological phenomenon [46,47]. Mixed pathology associations were not feasible due to the small TDP-43-pathology-defined cohort, thus warranting larger cohorts in the future. Although multiplex immunofluorescence with several neuronal subtype markers and 3D AI-assisted quantification provide high cellular resolution, they cannot fully resolve the temporal sequence of neuronal subtype degeneration. Another limitation is that TH labeling illustrates both dopaminergic and noradrenergic neuronal subtypes. Future work incorporating single-cell transcriptomics and spatial proteomics will be essential to determine whether TDP-43 accumulation precedes or follows interneuron dysfunction and to identify molecular pathways that confer selective vulnerability [66,67].

## 5. Conclusions

Together, these findings reveal that TDP-43 accumulation is highly cell-type-specific, preferentially coinciding with DβH-positive neurons across the limbic system. This selectivity may contribute to a unifying framework linking TDP-43 pathology to modulatory circuit breakdown, noradrenergic failure, and cognitive decline. By defining the cellular substrates of TDP-43 accumulation, this work lays the foundation for future mechanistic studies and may highlight potential therapeutic targets aimed at restoring noradrenergic balance in AD and PART.

## Figures and Tables

**Table 1:** Patient demographics.

Case. No.	Biological Sex	Age at Death (Years)	CERAD dx	CERAD Score	Thal Phase	Braak Stage	HpScl	ADNC	TDP-43 (LATE) Stage	Final Diagnosis
1	M	91	Def.	3	5	VI	-	A3B3C3	2	hAD
2	M	87	Moderate	2	5	V	-	A3B3C2	2	hAD
3	M	94	Def.	3	4	V	-	A3B3C3	2	hAD
4	F	93	Def.	3	5	V	-	A3B3C3	2	hAD
5	M	99	Moderate	2	5	V	-	A3B3C2	2	hAD
6	M	89	Def.	3	5	VI	+	A3B3C3	2	hAD
7	M	93	Def.	3	3	VI	+	A3B3C3	3	hAD
8	M	96	Moderate	2	4	IV	+	A3B2C2	2	hAD
9	F	93	Def.	3	4	V	-	A3B3C3	2	hAD
10	M	86	None	0	1	II	+	A1B1C0	2	Possible PART
11	F	93	None	0	0	II	-	A0B1C0	2	Definite PART
12	F	97	None	0	0	IV	+	A0B2C0	2	Definite PART
13	F	81	None	0	0	0	-	A0B0C0	0	Control
14	M	68	None	0	0	0	-	A0B0C0	0	Control
15	M	56	None	0	0	0	-	A0B0C0	0	Control

Demographic characteristics of each patient from this study. Acronyms: CERAD: Consortium to Establish a Registry for Alzheimer’s Disease; ADNC: Alzheimer’s disease neuropathologic change; TDP: transactive response DNA-binding protein 43; LATE: limbic-predominant age-related TDP-43 encephalopathy; h-AD: high likelihood of Alzheimer’s disease; PART: primary age-related tauopathy; HpScl: hippocampal sclerosis.

**Table 2:** Patient clinical test scores.

Case No.	Biological Sex	Age at Death (Years)	Education (Years)	APOE ALLELE-1	APOE ALLELE-2	APOE ε4	MMSE	CDRSUM	BNT	Trail A	Trail B
1	M	91	16	3	4	1	28	0.5	56	16	61
2	M	87	20	N/A	N/A	N/A	N/A	N/A	N/A	N/A	N/A
3	M	94	16	3	3	0	28	0	N/A	N/A	N/A
4	F	93	12	3	4	1	N/A	7	N/A	N/A	N/A
5	M	99	16	3	4	1	29	0	52	40	103
6	M	89	20	3	4	1	21	8	54	64	N/A
7	M	93	12	3	3	0	28	0	56	64	163
8	M	96	20	3	3	0	27	N/A	56	29	125
9	F	93	12	3	3	0	29	0	56	39	122
10	M	86	14	3	3	0	28	N/A	N/A	N/A	N/A
11	F	93	16	3	3	0	30	0	60	48	60
12	F	97	13	3	3	0	23	0.5	53	118	254
13	F	81	13	3	3	0	29	0	N/A	N/A	N/A
14	M	68	16	2	2	0	28	0	59	23	56
15	M	56	14	3	4	1	29	0	59	33	77

Demographic characteristics of each patient from this study. Acronyms: APOE ε4: Apolipoprotein E Epsilon-4; CDRSUM: Clinical Dementia Rating Scale score sum of boxes; BNT: Boston Naming Test.

## Data Availability

All aggregated data is available in the main text and figures or the Supplementary Materials. Primary data (e.g., images and spreadsheets) will be provided upon written request to the corresponding author. This paper does not report any original code. Any additional information can also be requested directly from the corresponding author.

## Supplementary Materials

The following supporting information can be downloaded at https://www.mdpi.com/article/10.3390/cells15201850/s1.

## Abbreviations

AI	Artificial Intelligence
AD	Alzheimer’s Disease
CA	Cornu Ammonis
ChAT	Choline Acetyltransferase
DβH	Dopamine β-Hydroxylase
ERC	Entorhinal Cortex
FTLD	Frontotemporal Lobar Degeneration
hAD	High-Likelihood AD
HCA	Hierarchical Cluster Analyses
LATE	Limbic-Predominant Age-Related TDP-43 Encephalopathy
NFT	Neurofibrillary Tangle
PART	Primary Age-Related Tauopathy
PCA	Principal Component Analysis
pTDP-43	Phosphorylated TDP-43
TDP-43	TAR DNA-Binding Protein of 43 kDa
TH	Tyrosine Hydroxylase
VGLUT1	Vesicular Glutamate Transporter-1
