# Supplementary material for: TDP-43 Coincides with Noradrenergic Neurons in Alzheimer’s Disease (AD) and Primary Age-Related Tauopathy (PART)
Source: Cells. 2026 Oct 12;15(20):1850. doi: 10.3390/cells15201850 (PMC13703715; doi:10.3390/cells15201850)
Supplement: Supplementary file 1 [file cells-15-01850-s001.zip › cells-4527208-supplementary.pdf]

SUPPLEMENTARY DATA

TDP-43 coincides with noradrenergic neurons in Alzheimer's disease (AD) and primary age related tauopathy (PART).

Gokhan Uruk, MD, PhD1*; Hossam Youssef, MBBCh1*; Rodolfo G. Gatto, MD, PhD1; Nadia Hossain, MBBS1; Jerusha Bhaskaran, MSc1; Yuqi Tian, PhD2; Mary M. Machulda3, PhD; Ronald C. Petersen, MD, PhD1; Jennifer L. Whitwell, PhD2; R. Ross Reichard, MD4, Keith A. Josephs, MD, MST, MSc1#

1Department of Neurology, Mayo Clinic, Rochester, MN, USA

2Department of Radiology, Mayo Clinic, Rochester, MN, USA

3Department of Neuropsychiatry (Neuropsychology), Mayo Clinic, Rochester, MN, USA

4Department of Laboratory Medicine and Pathology (Neuropathology), Mayo Clinic, Rochester, MN, USA.

*Authors contributed equally to this work.

Supp. Fig. 1.

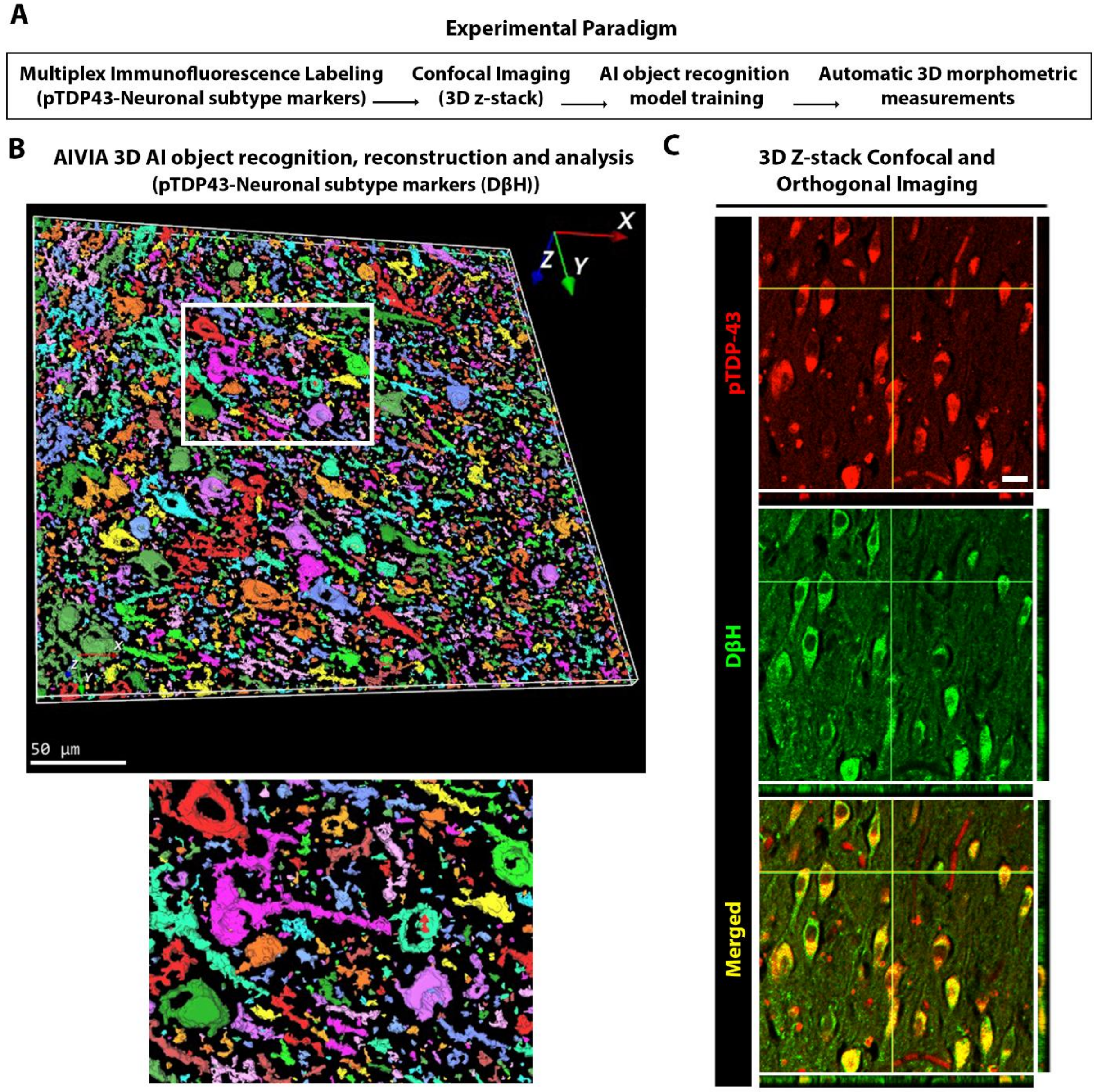

Supplementary Figure S1. Multiplex immunofluorescence, Z-stack confocal microscopy, AIVIA 3D object recognition, reconstruction, and analysis paradigm. (A) Schematic experimental diagram illustrating the experimental paradigm workflow. (B) 3D rendering of pTDP-43/Neuronal subtype specific (DβH) immunofluorescent staining of representative PART-TDP case from CA2/3. Each detected 3D particle automatically pseudo colored to demonstrate their spatial relationship in x-y-z axes. White rectangle shows where the insert is taken. Scale bars = 50 µm. (C) The representative maximally projected 2D, orthogonal x and y plane imaging results of PART-TDP with pTDP43 (red), DβH (green), and merged (yellow) were shown to point out the representative colocalizations in x-y-z axes. Scale bar = 20 µm

Supp. Fig. 2

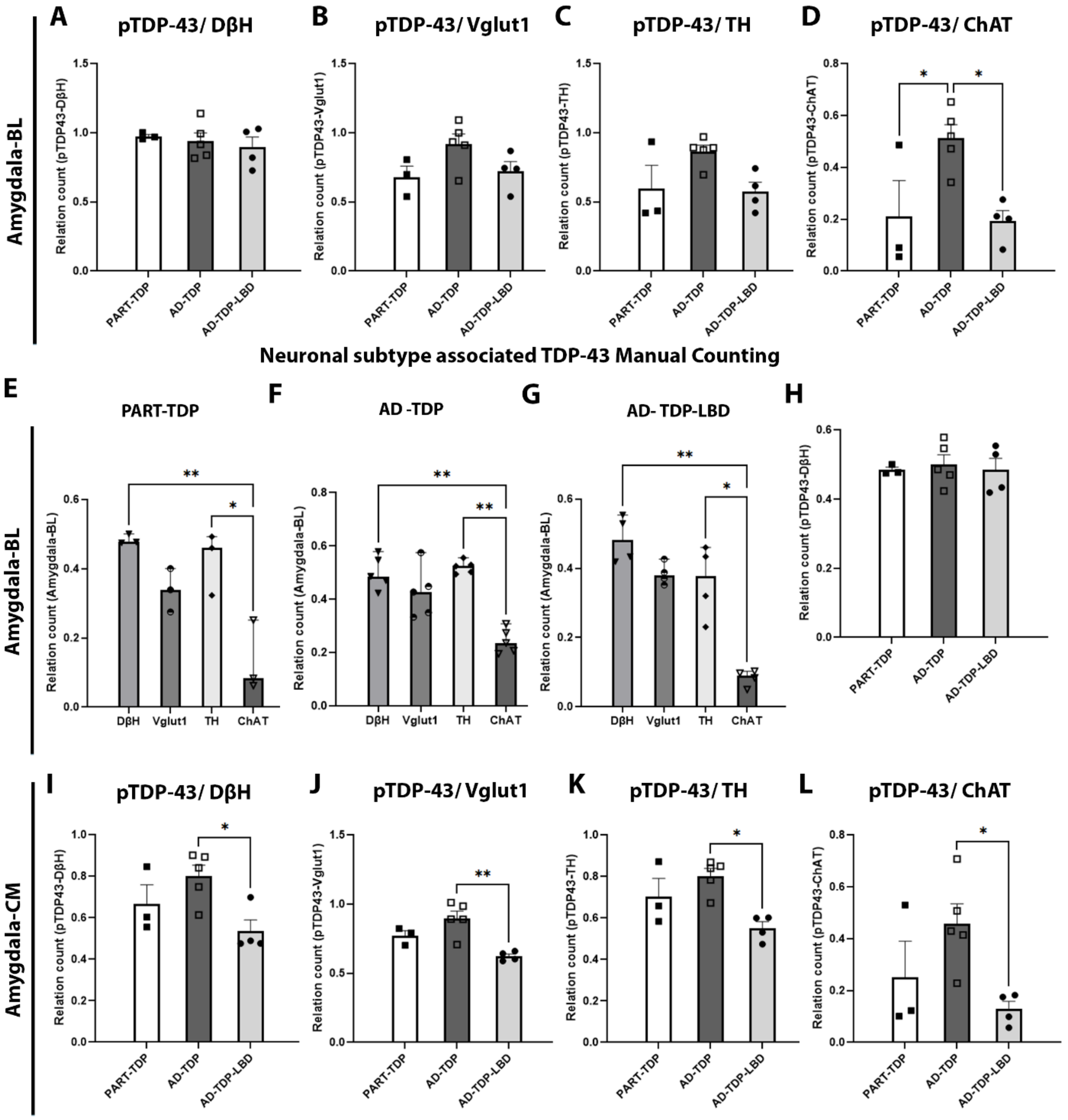

Supplementary Figure S2: Related to Figure 1 and 2. Comparative TDP-43 and in Noradrenergic, Dopaminergic, Cholinergic neurons, and Glutamatergic terminals in amygdala. AI segmentation and recognition of 3D objects from confocal z-stack images taken from pTDP-43, DβH, Vglut1, TH and ChAT in cases with PART-TDP, AD-TDP, AD-TDP-LBD. The relation counts between (A) pTDP-43 and DβH, (B) Vglut1, (C) TH, and (D) ChAT in amygdala-BL are shown. Neuronal subtype-associated TDP-43 colocalizations were also manually counted to validate AI classifiers and segmentation algorithm in E-H. The relation counts in amygdala-CM are assessed in I-L. * $p<0.05$, Kruskal-Wallis for grouped variables, Mann Whitney U for pairwise comparisons.

Supp. Fig. 3

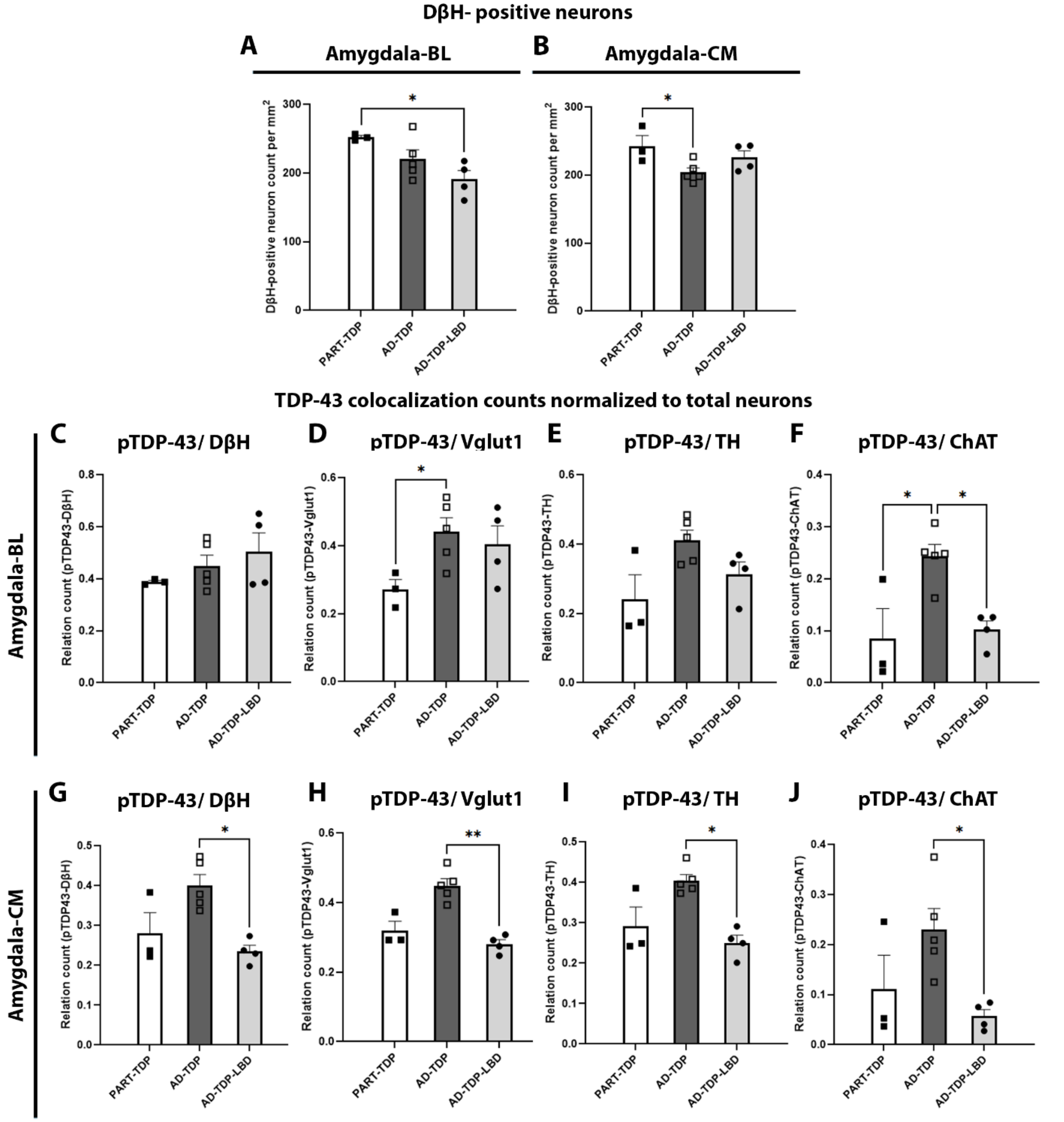

Supplementary Figure S3. Related to Figure 1, 2, and Supplementary Figure 2. DβH-positive neuron counts and normalized comparative TDP-43 and in Noradrenergic, Dopaminergic, Cholinergic neurons, and Glutamatergic terminals in amygdala. DβH-positive neurons counted per mm^2 in (A) Amygdala-BL, and (B) Amygdala-CM. AI segmentation and recognition of 3D objects from confocal z-stack images taken from pTDP-43, DβH, Vglut1, TH and ChAT in cases with PART-TDP, AD-TDP, AD-TDP-LBD. The relation counts between (C) pTDP-43 and DβH, (D) Vglut1, (E) TH, and (F) ChAT in amygdala-BL are shown. The relation counts in amygdala-CM are assessed in G-J. * $p<0.05$, Kruskal-Wallis for grouped variables, Mann Whitney U for pairwise comparisons.

Supp. Fig. 4

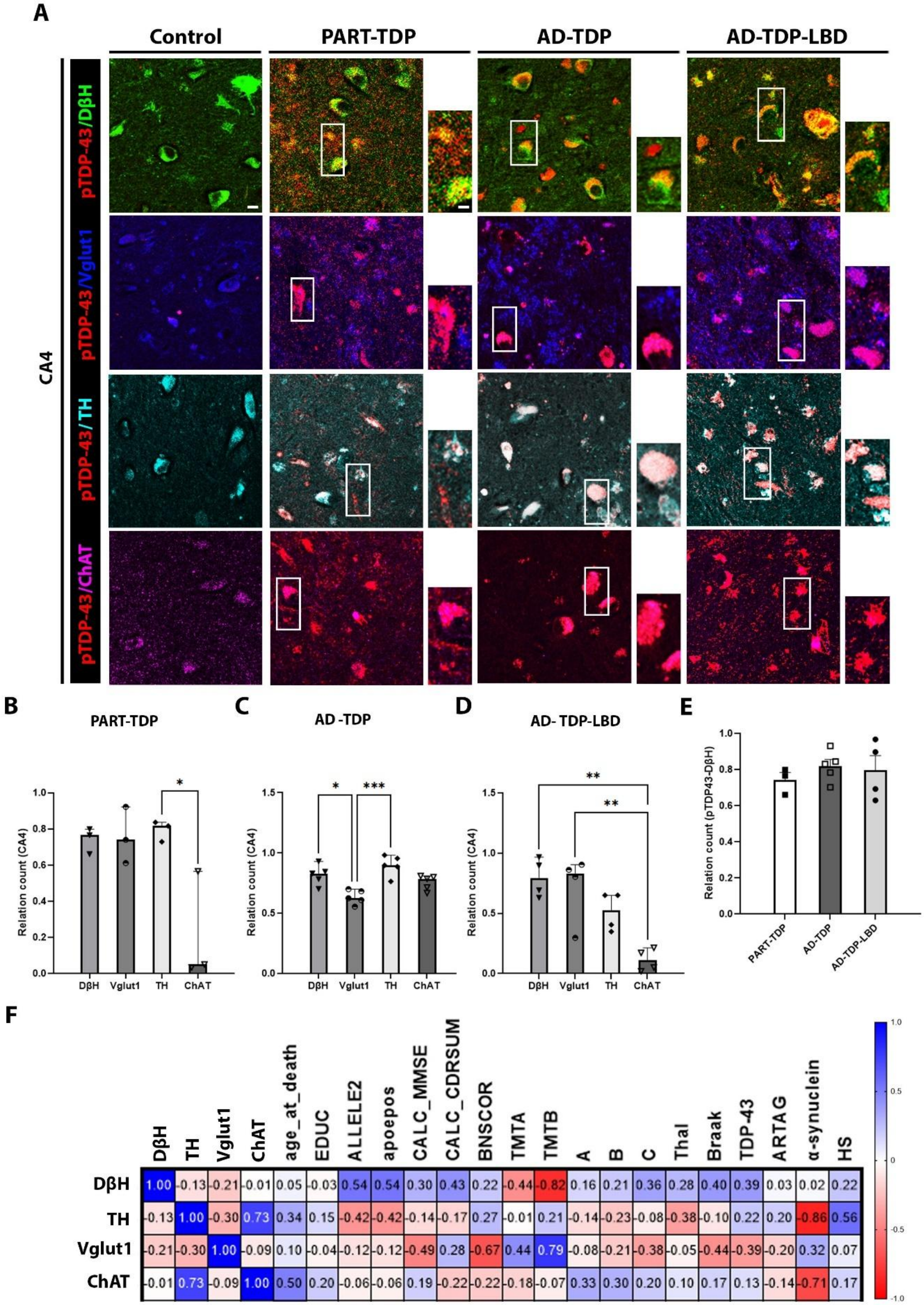

A
Control
PART-TDP
AD-TDP
AD-TDP-LBD
CA4
pTDP-43/DβH
pTDP-43/Vglut1
pTDP-43/TH
pTDP-43/ChAT
B
PART-TDP
Relation count (CA4)
DβH Vglut1 TH ChAT
C
AD -TDP
Relation count (CA4)
DβH Vglut1 TH ChAT
D
AD- TDP-LBD
Relation count (CA4)
DβH Vglut1 TH ChAT
E
Relation count (pTDP43-DβH)
PART-TDP
AD-TDP
AD-TDP-LBD
F
DβH TH Vglut1 ChAT age_at_death EDUC ALLELE2 apoepos CALC_MMSE CALC_CDRSUM BNSCOR TMTA TMTB A B C Thal Braak TDP-43 ARTAG α-synuclein HS
DβH 1.00 -0.13 -0.21 -0.01 0.05 -0.03 0.54 0.54 0.30 0.43 0.22 -0.44 -0.82 0.16 0.21 0.36 0.28 0.40 0.39 0.03 0.02 0.22
TH -0.13 1.00 -0.30 0.73 0.34 0.15 -0.42 -0.42 -0.14 -0.17 0.27 -0.01 0.21 -0.14 -0.23 -0.08 -0.38 -0.10 0.22 0.20 -0.86 0.56
Vglut1 -0.21 -0.30 1.00 -0.09 0.10 -0.04 -0.12 -0.12 -0.49 0.28 -0.67 0.44 0.79 -0.08 -0.21 -0.38 -0.05 -0.44 -0.39 -0.20 0.32 0.07
ChAT -0.01 0.73 -0.09 1.00 0.50 0.20 -0.06 -0.06 0.19 -0.22 -0.22 -0.18 -0.07 0.33 0.30 0.20 0.10 0.17 0.13 -0.14 -0.71 0.17

Supplementary Figure S4: TDP-43 accumulation in Noradrenergic, Dopaminergic, Cholinergic neurons, and Glutamatergic terminals in CA4. (A) Immunofluorescent staining of the controls, PART-TDP, AD-TDP and AD-TDP-LBD with pTDP-43 (red), DβH (green), Vglut1 (blue), TH (cyan), and ChAT (magenta). Each row represents merged images of pTDP-43 and DβH, Vglut1, TH and ChAT to demonstrate spatial relationship of pTDP-43 aggregates in each condition. White rectangles show where the inserts are taken. Scale bars = 20 µm. AI segmentation and recognition of 3D objects from confocal z-stack in each staining are performed. The relation counts between pTDP-43 and all markers are compared in (B) PART-TDP, (C) AD-TDP and (D) AD-TDP-LBD cases. (E) Relation count comparison of DβH (pTDP43- DβH) as the most differentiating, non-inhibitory marker in each group. (F) Representative Spearman correlation heatmap with respective coefficients between relation counts and clinicopathological assessments. Red and blue denote negative and positive correlations. * $p<0.05$, ** $p<0.01$, *** $p<0.001$, Kruskal-Wallis for grouped variables, Mann Whitney U for pairwise comparisons.

Supp. Fig. 5

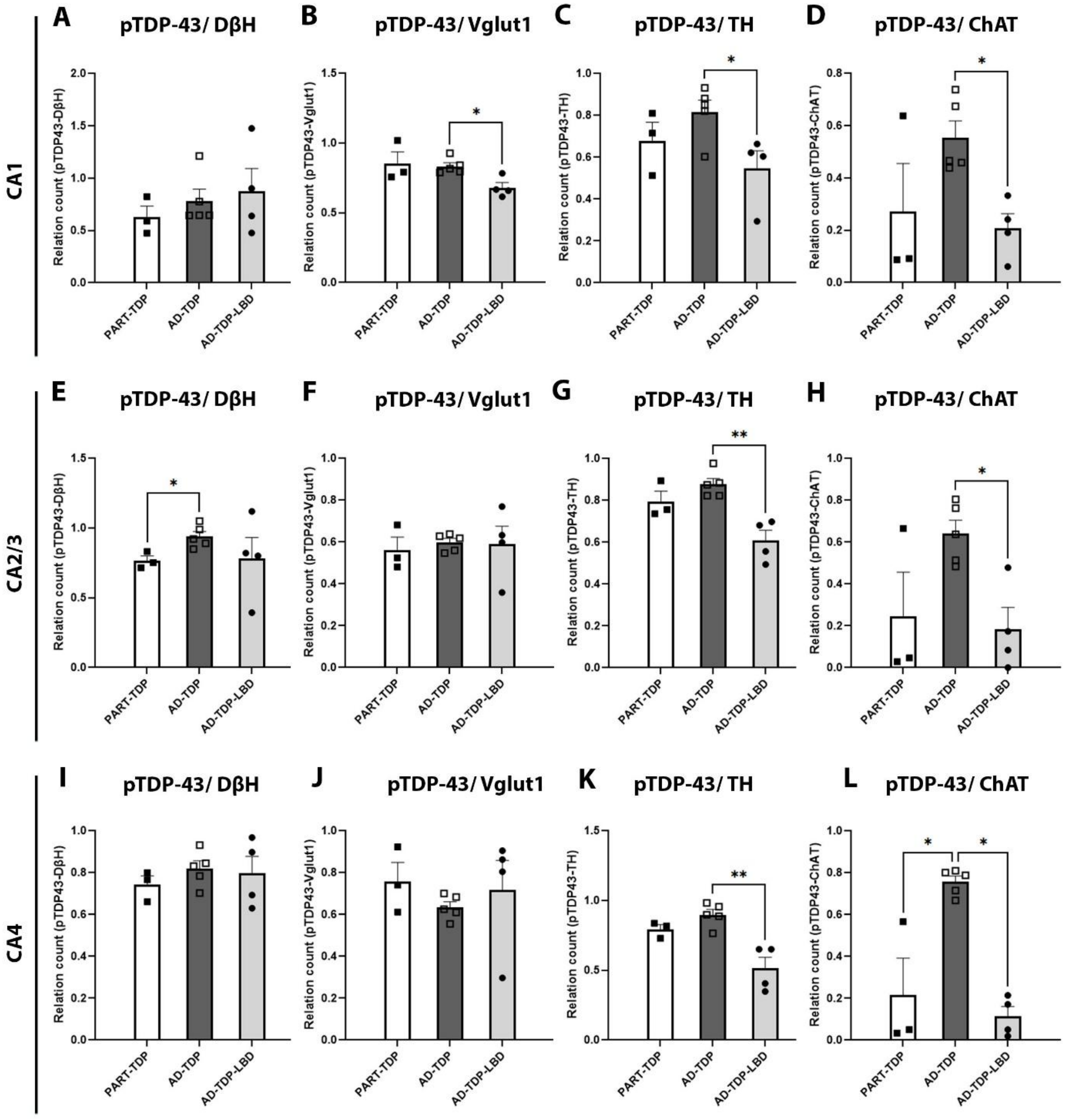

Supplementary Figure S5: Related to Figure 3, 4 and Supplementary Figure 2. Comparative TDP-43 in Noradrenergic, Dopaminergic, Cholinergic neurons, and Glutamatergic terminals in CA1, CA2/3, CA4 of Hippocampus. AI segmentation and recognition of 3D objects from confocal z-stack images taken from pTDP-43, DβH, Vglut1, TH and ChAT in cases with PART-TDP, AD-TDP, AD-TDP-LBD. The relation counts between (A) pTDP-43 and DβH, (B) Vglut1, (C) TH, and (D) ChAT in CA1 are shown. The relation counts in in CA2/3 are assessed in E-H while CA4 is demonstrated in I-L. . * $p<0.05$, ** $p<0.01$, Kruskal-Wallis for grouped variables, Mann Whitney U for pairwise comparisons.

Supp. Fig. 6

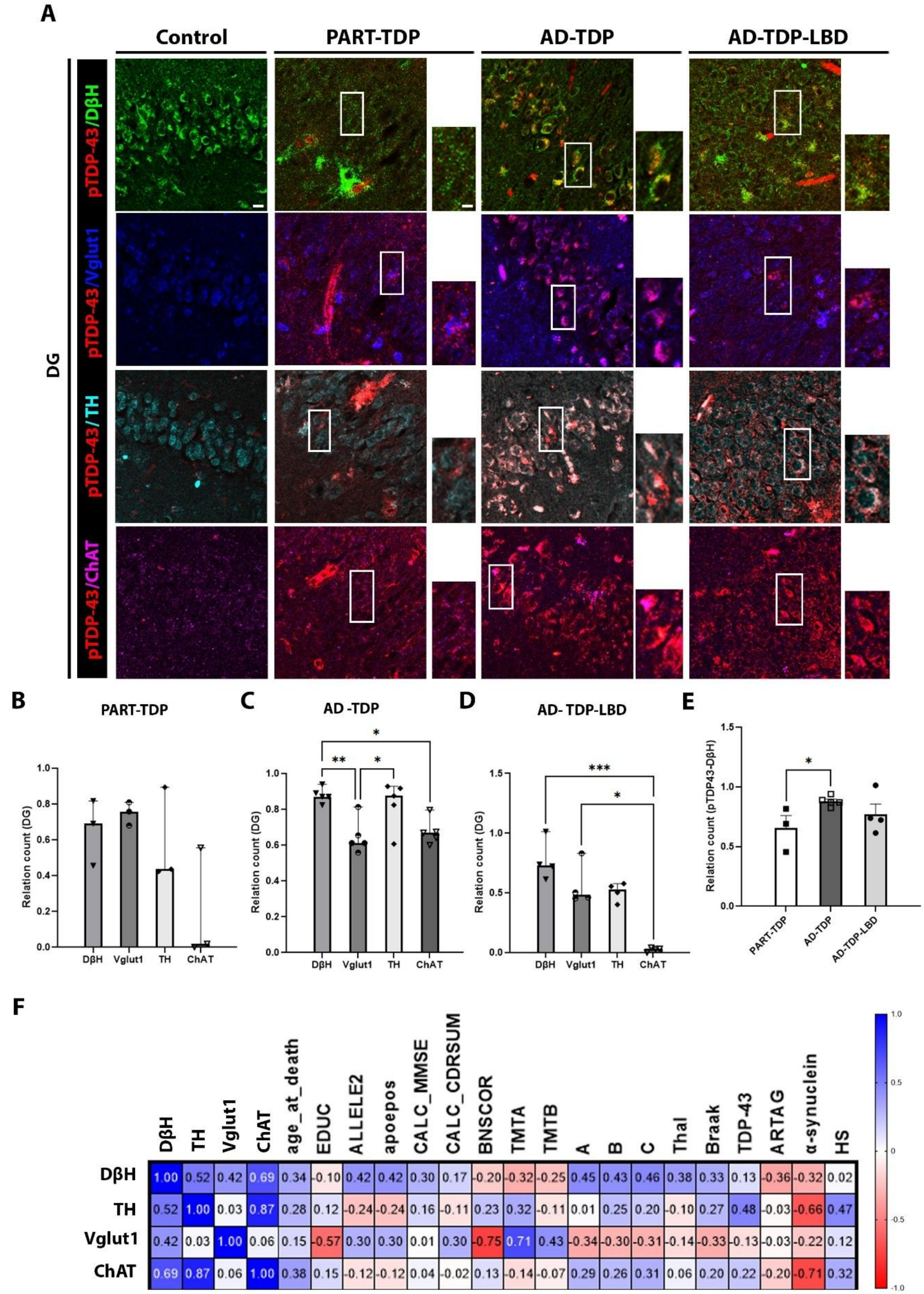
A
Control
PART-TDP
AD-TDP
AD-TDP-LBD
DG
pTDP-43/DβH
pTDP-43/Vglut1
pTDP-43/TH
pTDP-43/ChAT
B
PART-TDP
Relation count (DG)
DβH Vglut1 TH ChAT
C
AD -TDP
Relation count (DG)
DβH Vglut1 TH ChAT
D
AD- TDP-LBD
Relation count (DG)
DβH Vglut1 TH ChAT
E
Relation count (pTDP43-DβH)
PART-TDP AD-TDP AD-TDP-LBD
F
DβH TH Vglut1 ChAT age_at_death EDUC ALLELE2 apoepos CALC_MMSE CALC_CDRSUM BNSCOR TMTA TMTB A B C Thal Braak TDP-43 ARTAG α-synuclein HS
DβH 1.00 0.52 0.42 0.69 0.34 -0.10 0.42 0.42 0.30 0.17 -0.20 -0.32 -0.25 0.45 0.43 0.46 0.38 0.33 0.13 -0.36 -0.32 0.02
TH 0.52 1.00 0.03 0.87 0.28 0.12 -0.24 -0.24 0.16 -0.11 0.23 0.32 -0.11 0.01 0.25 0.20 -0.10 0.27 0.48 -0.03 -0.66 0.47
Vglut1 0.42 0.03 1.00 0.06 0.15 -0.57 0.30 0.30 0.01 0.30 -0.75 0.71 0.43 -0.34 -0.30 -0.31 -0.14 -0.33 -0.13 -0.03 -0.22 0.12
ChAT 0.69 0.87 0.06 1.00 0.38 0.15 -0.12 -0.12 0.04 -0.02 0.13 -0.14 -0.07 0.29 0.26 0.31 0.06 0.20 0.22 -0.20 -0.71 0.32
1.0 0.5 0 -0.5 -1.0

Supplementary Figure S6: TDP-43 accumulation in Noradrenergic, Dopaminergic, Cholinergic neurons, and Glutamatergic terminals in DG. (A) Immunofluorescent staining of the controls, PART-TDP, AD-TDP and AD-TDP-LBD with pTDP-43 (red), DβH (green), Vglut1 (blue), TH (cyan), and ChAT (magenta). Each row represents merged images of pTDP-43 and DβH, Vglut1, TH and ChAT to demonstrate spatial relationship of pTDP-43 aggregates in each condition. White rectangles show where the inserts are taken. Scale bars = 20 μm. AI segmentation and recognition of 3D objects from confocal z-stack in each staining are performed. The relation counts between pTDP-43 and all markers are compared in (B) PART-TDP, (C) AD-TDP and (D) AD-TDP-LBD cases. (E) Relation count comparison of DβH (pTDP43- DβH) as the most differentiating, non-inhibitory marker in each group. (F) Representative Spearman correlation heatmap with respective coefficients between relation counts and clinicopathological assessments. Red and blue denote negative and positive correlations. * $p<0.05$, ** $p<0.01$, Kruskal-Wallis for grouped variables, Mann Whitney U for pairwise comparisons.

Supp. Fig. 7

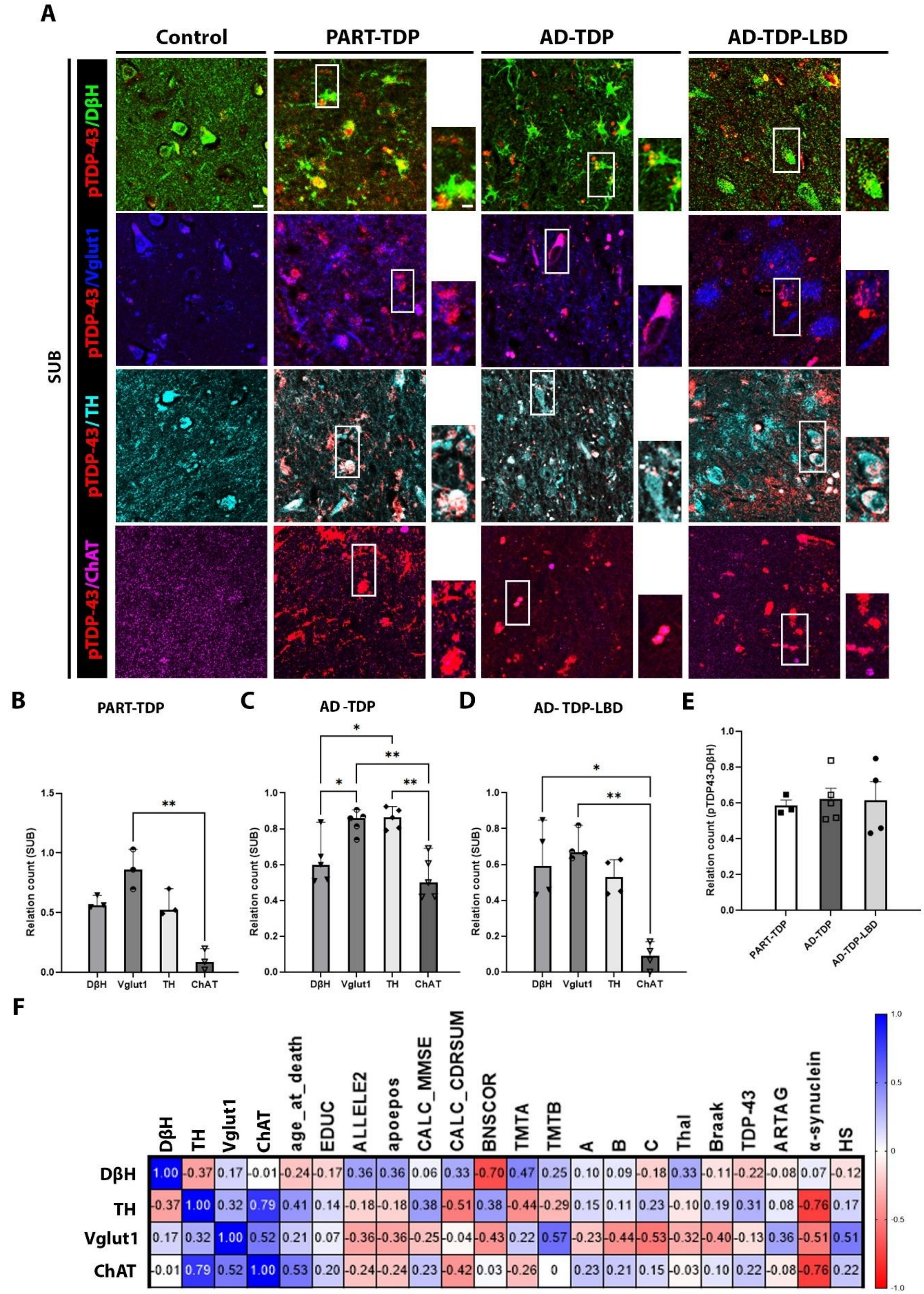
A
Control
PART-TDP
AD-TDP
AD-TDP-LBD
SUB
pTDP-43/DβH
pTDP-43/Vglut1
pTDP-43/TH
pTDP-43/ChAT
B
PART-TDP
Relation count (SUB)
DβH Vglut1 TH ChAT
C
AD -TDP
D
AD- TDP-LBD
E
Relation count (pTDP43-DβH)
PART-TDP AD-TDP AD-TDP-LBD
F
DβH TH Vglut1 ChAT age_at_death EDUC ALLELE2 apoepos CALC_MMSE CALC_CDRSUM BNSCOR TMTA TMTB A B C Thal Braak TDP-43 ARTAG α-synuclein HS
DβH 1.00 -0.37 0.17 -0.01 -0.24 -0.17 0.36 0.36 0.06 0.33 -0.70 0.47 0.25 0.10 0.09 -0.18 0.33 -0.11 -0.22 -0.08 0.07 -0.12
TH -0.37 1.00 0.32 0.79 0.41 0.14 -0.18 -0.18 0.38 -0.51 0.38 -0.44 -0.29 0.15 0.11 0.23 -0.10 0.19 0.31 0.08 -0.76 0.17
Vglut1 0.17 0.32 1.00 0.52 0.21 0.07 -0.36 -0.36 -0.25 -0.04 -0.43 0.22 0.57 -0.23 -0.44 -0.53 -0.32 -0.40 -0.13 0.36 -0.51 0.51
ChAT -0.01 0.79 0.52 1.00 0.53 0.20 -0.24 -0.24 0.23 -0.42 0.03 -0.26 0 0.23 0.21 0.15 -0.03 0.10 0.22 -0.08 -0.76 0.22

Supplementary Figure S7: TDP-43 accumulation in Noradrenergic, Dopaminergic, Cholinergic neurons, and Glutamatergic terminals in SUB. (A) Immunofluorescent staining of the controls, PART-TDP, AD-TDP and AD-TDP-LBD with pTDP-43 (red), DβH (green), Vglut1 (blue), TH (cyan), and ChAT (magenta). Each row represents merged images of pTDP-43 and DβH, Vglut1, TH and ChAT to demonstrate spatial relationship of pTDP-43 aggregates in each condition. White rectangles show where the inserts are taken. Scale bars = 20 µm. AI segmentation and recognition of 3D objects from confocal z-stack in each staining are performed. The relation counts between pTDP-43 and all markers are compared in (B) PART-TDP, (C) AD-TDP and (D) AD-TDP-LBD cases. (E) Relation count comparison of DβH (pTDP43- DβH) as the most differentiating, non-inhibitory marker in each group. (F) Representative Spearman correlation heatmap with respective coefficients between relation counts and clinicopathological assessments. Red and blue denote negative and positive correlations. * $p<0.05$, ** $p<0.01$, *** $p<0.001$, Kruskal-Wallis for grouped variables, Mann Whitney U for pairwise comparisons.

Supp. Fig. 8

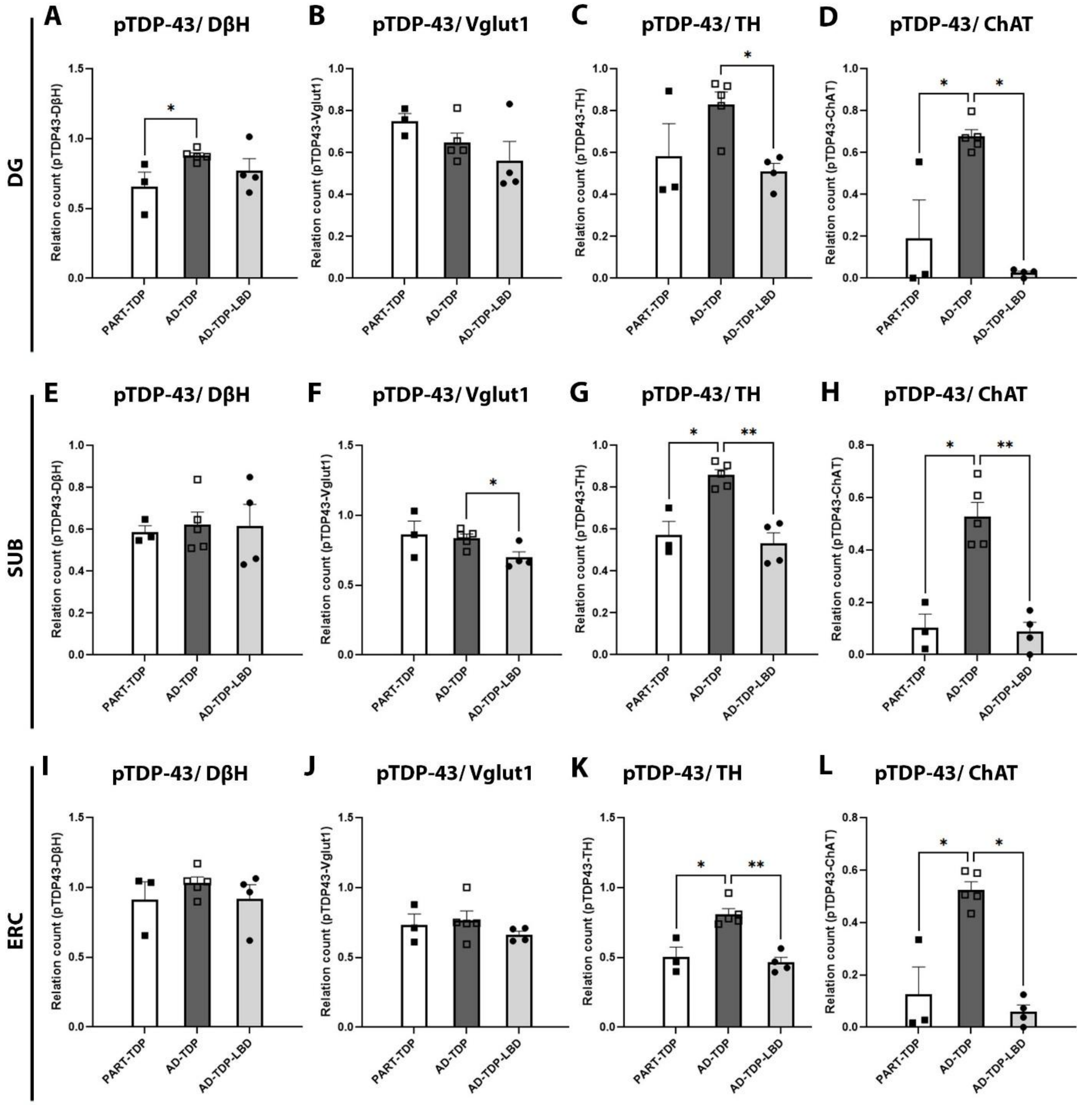

Supplementary Figure S8: Related to Supplementary Figure 4, 5 and Figure 5. Comparative TDP-43 in Noradrenergic, Dopaminergic, Cholinergic neurons, and Glutamatergic terminals in DG, SUB and ERC. AI segmentation and recognition of 3D objects from confocal z-stack images taken from pTDP-43, DβH, Vglut1, TH and ChAT in cases with PART-TDP, AD-TDP, AD-TDP-LBD. The relation counts between (A) pTDP-43 and DβH, (B) Vglut1, (C) TH, and (D) ChAT in DG are shown. The relation counts in SUB are assessed in E-H while ERC is demonstrated in I-L. * $p<0.05$, ** $p<0.01$, Kruskal-Wallis for grouped variables, Mann Whitney U for pairwise comparisons.

Supp. Fig. 9

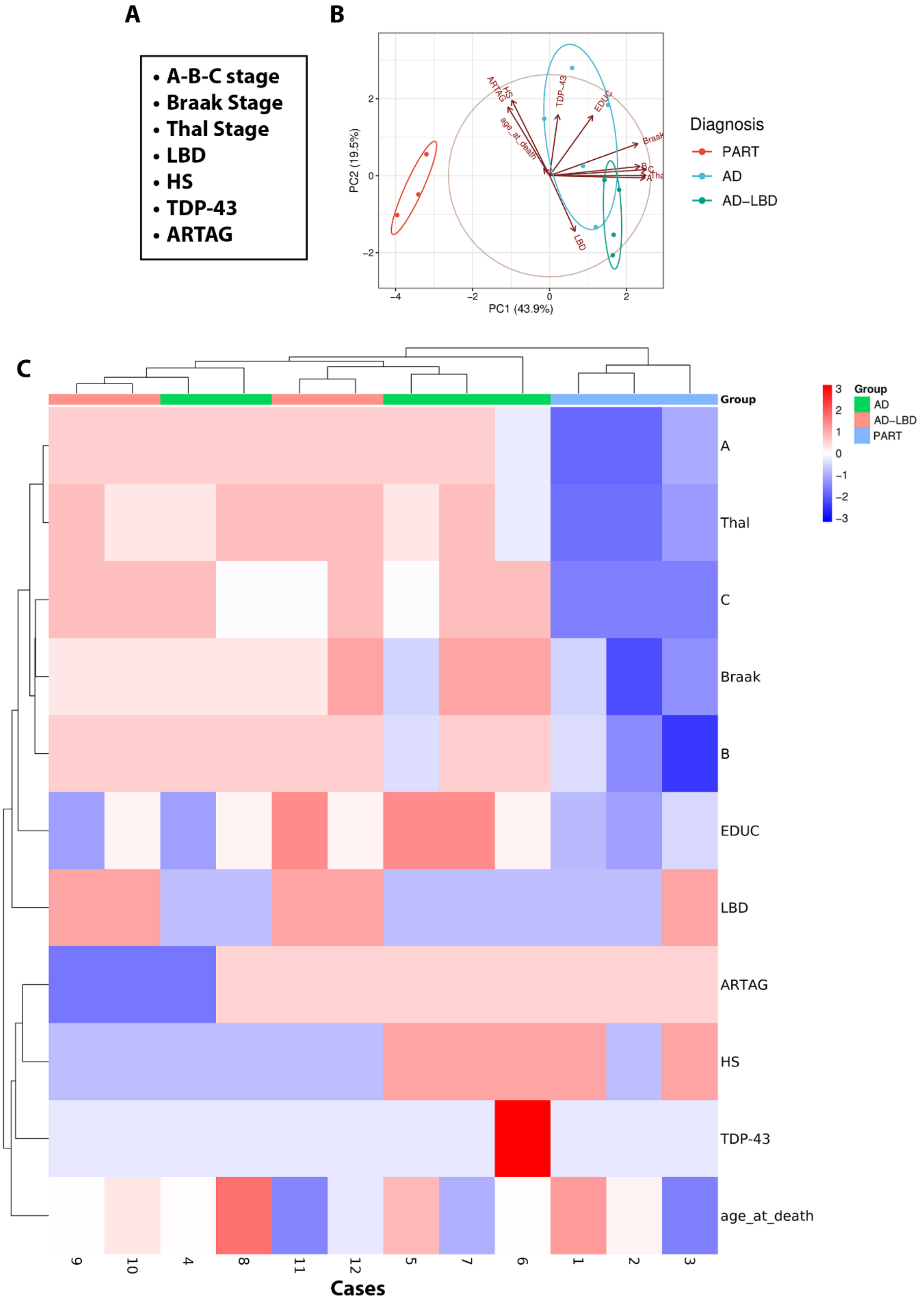
A
• A-B-C stage
• Braak Stage
• Thal Stage
• LBD
• HS
• TDP-43
• ARTAG
B
PC1 (43.9%)
PC2 (19.5%)
Diagnosis
PART
AD
AD−LBD
C
Group
AD
AD−LBD
PART
A
Thal
C
Braak
B
EDUC
LBD
ARTAG
HS
TDP-43
age_at_death
Cases

Supplementary Figure S9. Related to Figure 6. Principal component (PCA) and hierarchical cluster analyses (HCA) of 12 cases using only neuropathological assessments. (A) Schematic of parameters of interest. (B) PCA analysis of the three distinct groups, PART-TDP, AD-TDP, and AD-TDP-LBD. n= 3-5 case/ group. Arrows indicate directionality of each parameter. Ellipsoids harbor cases belonging to the same neuropathological diagnostic group. PCA circle shows the correlation via maximal possible variance. (C) Hierarchical cluster analysis with Euclidean Ward method of 12 cases. Dendrograms showing the proximity of each case and group. Green; AD-TDP, orange: AD-TDP-LBD, blue: PART-TDP. Z scores between -3 and 3 are shown as blue to red spectrum.

Supp. Fig. 10

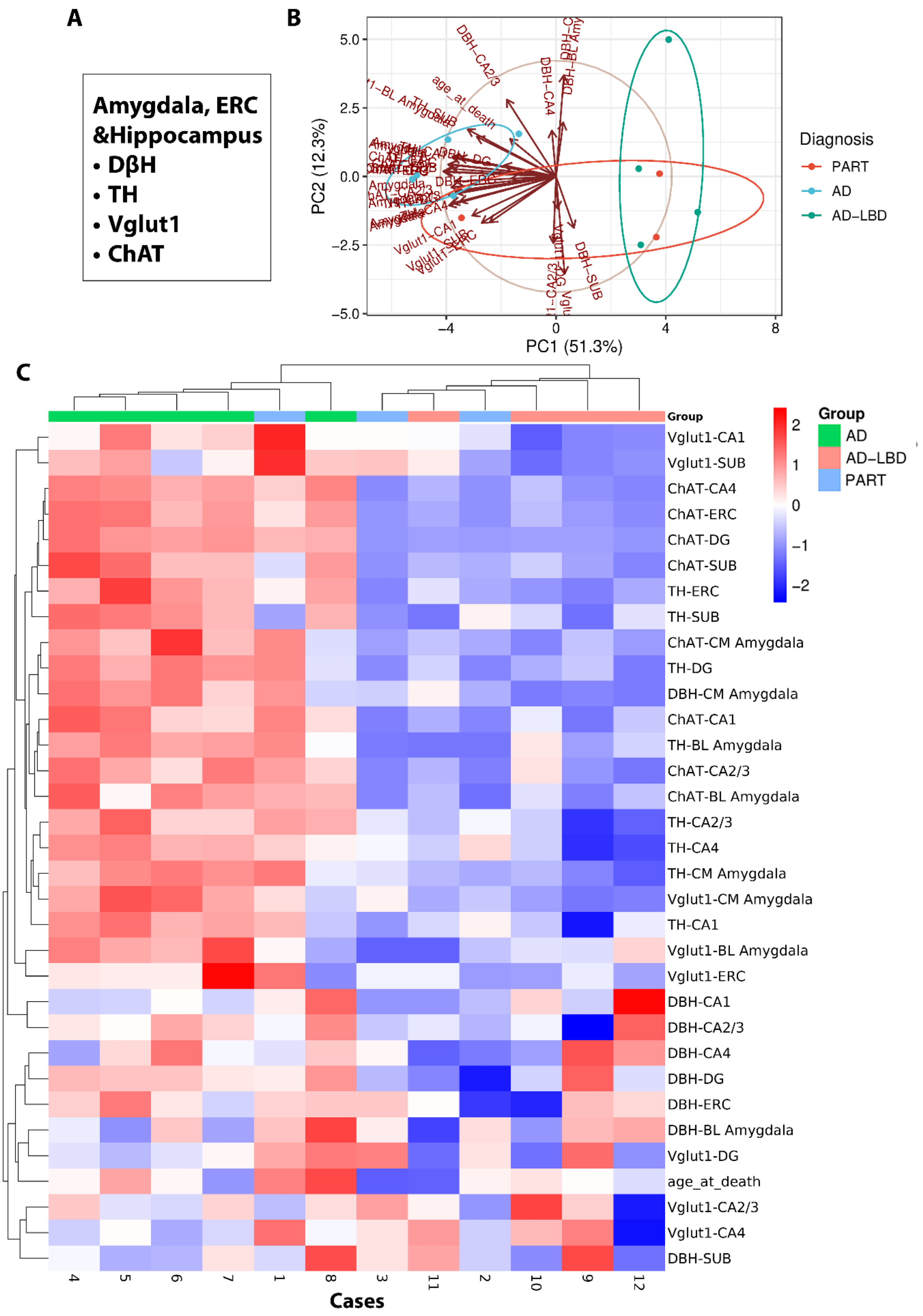
A
Amygdala, ERC &Hippocampus
• DβH
• TH
• Vglut1
• ChAT
B
PC2 (12.3%)
PC1 (51.3%)
5.0
2.5
0.0
−2.5
−5.0
−4
0
4
8
DBH-CA2/3
DBH-CA4
DBH-SUB
age_at_death
Diagnosis
PART
AD
AD−LBD
C
Group
Vglut1-CA1
Vglut1-SUB
ChAT-CA4
ChAT-ERC
ChAT-DG
ChAT-SUB
TH-ERC
TH-SUB
ChAT-CM Amygdala
TH-DG
DBH-CM Amygdala
ChAT-CA1
TH-BL Amygdala
ChAT-CA2/3
ChAT-BL Amygdala
TH-CA2/3
TH-CA4
TH-CM Amygdala
Vglut1-CM Amygdala
TH-CA1
Vglut1-BL Amygdala
Vglut1-ERC
DBH-CA1
DBH-CA2/3
DBH-CA4
DBH-DG
DBH-ERC
DBH-BL Amygdala
Vglut1-DG
age_at_death
Vglut1-CA2/3
Vglut1-CA4
DBH-SUB
2
1
0
−1
−2
Group
AD
AD−LBD
PART
4
5
6
7
1
8
3
11
2
10
9
12
Cases

Supplementary Figure S10. Related to Figure 7. PCA and HCA of 12 cases using only pTDP-43 colocalization relation counts. (A) Schematic of parameters of interest. (B) PCA analysis of the three distinct groups, PART-TDP, AD-TDP, and AD-TDP-LBD. n= 3-5 case/ group. Arrows indicate directionality of each parameter. Ellipsoids harbor cases belonging to the same neuropathological diagnostic group. PCA circle shows the correlation via maximal possible variance. (C) HCA with Euclidean Ward method of 12 cases. Dendrograms showing the proximity of each case and group. Green; AD-TDP, orange: AD-TDP-LBD, blue: PART-TDP. Z scores between -2 and 2 are shown as blue to red spectrum.
